# Screening-Identified Oxazole-4-Carboxamide KB-2777 Exhibits In Vitro Anti-Coronavirus Activity

**DOI:** 10.3390/pharmaceutics17111477

**Published:** 2025-11-16

**Authors:** Bud Jung, Woonsung Na, Minjoo Yeom, Jong-Woo Lim, Hai Quynh Do, Geonhee Jang, Min-A Ban, Ji-eun Yang, Youngjoo Byun, Daesub Song

**Affiliations:** 1 Department of Pharmacy, College of Pharmacy, Korea University, Sejong 30019, Republic of Korea; 2 Department of Oral Microbiology and Immunology, School of Dentistry, Seoul National University, Seoul 08826, Republic of Korea; 3 Department of Virology, College of Veterinary Medicine and Research Institute for Veterinary Science, Seoul National University, Seoul 08826, Republic of Korea; 4 Interdisciplinary Major Program in Innovative Pharmaceutical Sciences, Korea University, Sejong 30019, Republic of Korea

**Keywords:** oxazole-4-carboxamide, HCoV-NL63, HCoV-OC43, PEDV, time-of-addition, combination therapy

## Abstract

**Background/Objectives:** Direct-acting antivirals vary by lineage and face rapid resistance. We identified the oxazole-4-carboxamide lead KB-2777 and aimed to define its in vitro activity across α/β-coronaviruses, time-of-addition (TOA) profile, host-response signatures, and combinability with benchmark DAAs. **Methods:** We tested KB-2777 (≤25 μM) against HCoV-NL63 (LLC-MK2), HCoV-OC43 (Vero E6; MRC-5 for transcript profiling), and PEDV (Vero E6). We quantified extracellular viral RNA by RT-qPCR at 72 h (*n* = 3) and confirmed activity by spike-protein immunofluorescence (IFA), cytopathic effect (CPE) protection, and TCID_50_. We compared TOA regimens (full, pre, co, post), evaluated combinations with nirmatrelvir (NL63) or GS-441524 (OC43) using ZIP scores, and profiled infection-context transcripts (IL6, IFNB1, ISG15, NRF2/antioxidant, UPR). **Results:** KB-2777 reduced viral RNA with EC_50_ 5.27 μM (NL63), 1.83 μM (OC43), and 1.59 μM (PEDV) without cytotoxicity in the tested range. In NL63 post-treatment, inhibition was minimal at 24 h but clear at 48–72 h (EC_50_ 2.42 μM at 48 h; 5.25 μM at 72 h). TCID_50_ decreased at 48 h (12.5–25 μM, *n* = 3, *p* < 0.0001), and IFA/CPE corroborated antiviral activity. TOA ranked full > pre ≈ post > co. Combinations were additive to synergistic (ZIP 5.16 with nirmatrelvir; 8.40 with GS-441524). In OC43-infected MRC-5 cells, KB-2777 attenuated IL6, IFNB1, ISG15, and selected UPR transcripts, with limited changes in uninfected cells (*n* = 3). **Conclusions:** KB-2777 shows reproducible cell-based anti-coronavirus activity across α/β lineages, a TOA signature consistent with early post-entry host modulation, and favorable, non-antagonistic combinability with DAAs. These findings support target deconvolution, SAR/ADME optimization, and evaluation in primary airway and in vivo models.

## 1. Introduction

Direct-acting antivirals (DAAs) that target viral proteins, such as the 3C-like protease (3CLpro) and RNA-dependent RNA polymerase (RdRp), engage a single molecular target [1,2]. Consequently, efficacy varies across lineages and variants, and resistance and cross-resistance emerge rapidly via substitutions in the targeted proteins [3,4,5,6,7]. Clinical responses in immunocompromised, high-risk populations remain inconsistent, and DAA monotherapy often lacks depth and durability. Within-class combinations are constrained by antagonism risk and low genetic barriers to resistance [8,9,10,11,12]. These limitations support the development of antivirals with distinct mechanisms of action that broaden lineage coverage and raise genetic barriers to resistance.

From a medicinal chemistry perspective, oxazole-4-carboxamides offer a dual opportunity that addresses these gaps. Oxazoles are privileged heteroaromatic scaffolds found across anti-infective and host-modulating chemotypes, and the heteroaryl-carboxamide linkage furnishes directional hydrogen-bonding capacity together with tunable electronic and polarity properties amenable to pharmacological optimization [13,14,15,16]. On the direct-acting side, oxazole- or imidazole-containing carboxamides have been reported as inhibitors of viral proteases, including 3CLpro enzymes, and carboxamide cores such as pyrazine-2-carboxamides (e.g., favipiravir) provide precedent for targeting RNA virus polymerases [17,18]. On the host-directed side, several oxazole derivatives modulate pathways implicated in coronavirus pathobiology, including nuclear factor κB (NF-κB)-driven cytokine induction, nuclear factor erythroid 2-related factor 2 (NRF2)-linked antioxidant responses, and endoplasmic reticulum (ER) stress and the unfolded protein response (UPR), suggesting the potential to temper infection-associated hyperinflammation without broad immunosuppression. Taken together, oxazole-4-carboxamides represent a rational starting point for efforts to broaden lineage coverage, mitigate antagonism risk in combinations, and raise genetic barriers to resistance by coupling direct and host-directed mechanisms.

Here, we synthesized a focused library of oxazole-4-carboxamide derivatives (Scheme S1) and systematically profiled in vitro activity against representative alphacoronaviruses, human coronavirus NL63 (HCoV-NL63) and porcine epidemic diarrhea virus (PEDV), and a betacoronavirus, human coronavirus OC43 (HCoV-OC43). Antiviral efficacy at 72 h was quantified by reverse transcription quantitative PCR (RT-qPCR) of culture supernatants and protection from cytopathic effect (CPE); for NL63, immunofluorescence assay (IFA) and TCID_50_ provided orthogonal confirmation. To contextualize the timing of action, we implemented a time-of-addition (TOA) framework (pre-, co- and post-infection and continuous dosing). In parallel, we assessed time-resolved mRNA changes using a targeted host-response transcript panel covering type I interferon and interferon-stimulated genes (IFN–ISG), NRF2-linked antioxidant responses, and the UPR. We also evaluated combinations with benchmark direct-acting antivirals (nirmatrelvir, GS-441524) to assess additivity or synergy. Our objectives were to (i) identify oxazole-4-carboxamides with reproducible anti-coronavirus activity and (ii) quantify TOA responses and host-response signatures to inform subsequent scaffold optimization (SAR/ADME) and mechanistic studies.

## 2. Materials and Methods

### 2.1. General Procedure for the Preparation of Compounds KB-2777

All reagents and analytical-grade solvents were purchased from commercial suppliers and used without further purification unless otherwise specified. Reaction progress was monitored by thin-layer chromatography (TLC) on silica gel 60F254 plates (Merck, Darmstadt, Germany) under UV light at 254 or 365 nm. ^1^H and ^13^C nuclear magnetic resonance (NMR) spectra were recorded at ambient temperature on a Bruker Ultrashield 600 MHz Plus spectrometer (Bruker, Bremen, Germany). Chemical shifts (δ) are reported in parts per million (ppm) relative to tetramethylsilane (TMS) or residual solvent peaks (CDCl_3_: δ = 7.26 for ^1^H; δ = 77.16 for ^13^C; DMSO-d_6_: δ = 2.50 for ^1^H; δ = 39.52 for ^13^C). Multiplicities are given as s (singlet), brs (broad singlet), d (doublet), t (triplet), q (quartet), m (multiplet), dd (doublet of doublets), etc. High-resolution mass spectra (HRMS) were obtained using an Agilent 6530 Accurate-Mass Q-TOF LC/MS system. The purity of all final compounds was measured by analytical reverse-phase high-performance liquid chromatography (RP-HPLC) on an Agilent 1260 Infinity system equipped with a Phenomenex C18 column (150 × 4.6 mm, 3 µm, 110 Å). RP-HPLC was performed using water (0.1% trifluoroacetic acid, TFA) and acetonitrile (0.1% TFA) as mobile phases under gradient conditions (50:50 to 90:10, *v*/*v*) at a flow rate of 1 mL/min. UV detection was carried out at 220 or 254 nm. All tested compounds were confirmed to have a purity of >95% (Figures S1–S13).

### 2.2. Compound KB-2777

**Scheme 1:**
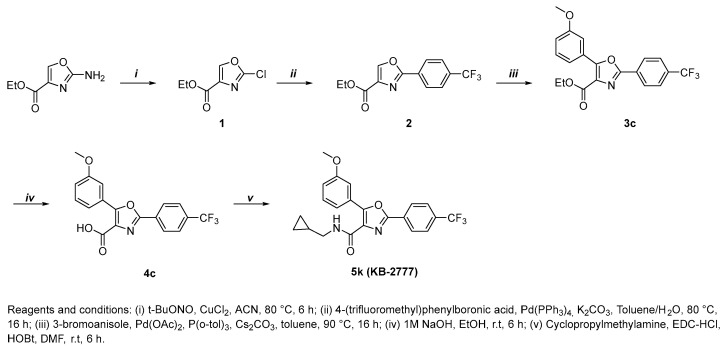
Chemical synthesis of KB-2777.

The target compound **5k** (KB-2777) was synthesized in a total of five steps, and the overall route is depicted in Scheme 1. Ethyl 2-aminooxazole-4-carboxylate was first subjected to diazotization followed by chlorination using tert-butyl nitrite and CuCl_2_ to afford compound **1**. Compound **1** was then converted to compound **2** via a Suzuki–Miyaura coupling reaction with 4-(trifluoromethyl)phenylboronic acid under Pd(PPh_3_)_4_ catalysis and basic conditions. Direct arylation of compound **2** with 3-bromoanisole was performed in the presence of Pd(OAc)_2_ and tri(o-tolyl)phosphine under basic, nonpolar solvent conditions to give compound **3c**. Hydrolysis of compound **3c** with aqueous NaOH solution yielded the corresponding carboxylic acid **4c**. Finally, amide coupling of compound **4c** with cyclopropylmethylamine mediated by EDC/HOBt afforded the target compound **5k** (KB-2777).

#### 2.2.1. Ethyl 2-Chlorooxazole-4-Carboxylate (**1**)

The compound was synthesized as previously described in the literature, and the obtained ^1^H NMR spectrum was consistent with the reported data [19].

#### 2.2.2. Ethyl 2-(4-(trifluoromethyl)phenyl)Oxazole-4-Carboxylate (**2**)

To a solution of compound **1** (358 mg, 2.04 mmol) and 4-(trifluoromethyl)phenylboronic acid (1.5 eq) in toluene, a solution of K_2_CO_3_ (2.0 eq) in H_2_O was added, followed by tetrakis(triphenylphosphine)palladium(0) (0.05 eq). The reaction mixture was stirred under argon at 80 °C for 16 h. After cooling to room temperature, the mixture was partitioned between ethyl acetate and water, and the aqueous layer was further extracted with ethyl acetate. The combined organic extracts were washed with 3 N HCl, dried over anhydrous MgSO_4_, and concentrated under reduced pressure. The crude residue was purified by column chromatography on silica gel (hexane/ethyl acetate = 9:1 → 6:1, *v*/*v*) to afford compound **2** as a white solid (302 mg, 1.06 mmol, 52%). R_f_ = 0.61 (hexane/ethyl acetate = 2:1, *v*/*v*). ^1^H NMR (600 MHz, CDCl_3_) *δ* 8.35 (s, 1H), 8.25 (d, *J* = 7.8 Hz, 2H), 7.75 (d, *J* = 8.4 Hz, 2H), 4.47 (q, *J* = 7.2 Hz, 2H), 1.44 (t, *J* = 7.1 Hz, 3H).

#### 2.2.3. Ethyl 5-(3-methoxyphenyl)-2-(4-(trifluoromethyl)phenyl)Oxazole-4-Carboxylate (**3c**)

A solution of compound **2** (300 mg, 0.78 mmol), 3-bromoanisole (1.2 eq), CS_2_CO_3_ (1.5 eq), tri(o-tolyl)phosphine (0.2 eq), and palladium acetate (0.2 eq) in toluene (20 mL) was stirred under argon at 90 °C for 16 h. After cooling to room temperature, the reaction mixture was partitioned between ethyl acetate, water, and 3 N HCl. The aqueous phase was extracted with ethyl acetate, and the combined organic layers were washed, dried over anhydrous MgSO_4_, and concentrated under reduced pressure. The residue was purified by silica gel column chromatography (hexane/ethyl acetate = 9:1 → 7:1, *v*/*v*) to afford compound **3c** as a white solid (154 mg, 0.39 mmol, 51%). R_f_ = 0.58 (hexane/ethyl acetate = 2:1, *v*/*v*). ^1^H NMR (600 MHz, CDCl_3_) *δ* 8.27 (d, *J* = 8.2 Hz, 2H), 7.75 (app, *J* = 8.2 Hz, 1H, overlapped with s, 1H), 7.68 (d, *J* = 7.8 Hz, 1H), 7.42 (dd, *J* = 7.8, 8.2 Hz, 1H), 7.04 (d, *J* = 8.2 Hz, 1H), 4.47 (q, *J* = 7.1 Hz, 2H), 3.90 (s, 3H), 1.43 (t, *J* = 7.1 Hz, 3H).

#### 2.2.4. 5-(3-methoxyphenyl)-2-(4-(trifluoromethyl)phenyl)Oxazole-4-Carboxylic Acid (**4c**)

A solution of compound **3c** (154 mg, 0.39 mmol) and 1 M NaOH (3.0 eq) in ethanol (30 mL) was stirred under argon at room temperature for 6 h. The solvent was removed under reduced pressure, and the residue was acidified with water and 3 N HCl to pH 1–2. The resulting precipitate was collected by filtration, washed with water, and dried to afford compound **4c** as a white solid (120 mg, 0.33 mmol, 85%). R_f_ = 0.45 (Dichloromethane/MeOH = 10:1, *v*/*v*). ^1^H NMR (600 MHz, DMSO-d_6_) *δ* 8.32 (d, *J* = 8.2 Hz, 2H), 7.96 (d, *J* = 8.2 Hz, 2H), 7.82–7.76 (m, 1H), 7.71 (d, *J* = 7.9 Hz, 1H), 7.48 (dd, *J* = 7.9, 8.3 Hz, 1H), 7.13 (d, *J* = 8.3 Hz, 1H), 3.84 (s, 3H).

#### 2.2.5. N-(cyclopropylmethyl)-5-(3-methoxyphenyl)-2-(4-(trifluoromethyl)phenyl)Oxazole-4-Carboxamide (**5k**, KB-2777)

To a stirred solution of compound **4c** (247 mg, 0.68 mmol), HOBt (1.6 eq), and EDC·HCl (1.6 eq) in DMF was added cyclopropylmethylamine (1.2 eq) at room temperature. The reaction mixture was stirred under argon at the same temperature until TLC analysis indicated complete conversion (typically 6 h). The mixture was extracted with ethyl acetate and water, and the organic layer was washed with brine, dried over anhydrous MgSO_4_, filtered, and concentrated under reduced pressure. The crude residue was purified by silica gel column chromatography (hexane/ethyl acetate = 7:1 → 2:1, *v*/*v*) to afford compound **5k** (241 mg, 0.58 mmol, 86%) as a white solid. R_f_ = 0.72 (hexane/ethyl acetate = 2:1, *v*/*v*). ^1^H NMR (600 MHz, CDCl_3_) δ 8.24 (d, *J* = 8.1 Hz, 2H), 8.18–8.13 (m, 1H), 7.93 (d, *J* = 8.3 Hz, 2H), 7.77 (d, *J* = 8.1 Hz, 2H), 7.49 (brs, 1H), 7.40 (dd, *J* = 7.8, 8.0 Hz, 1H), 7.00 (d, *J* = 8.0 Hz, 1H), 3.92 (s, 3H), 3.39–3.32 (m, 2H), 1.16–1.07 (m, 1H), 0.63–0.56 (m, 2H), 0.35–0.29 (m, 2H). ^13^C NMR (151 MHz, CDCl_3_) *δ* 161.04, 159.71, 157.00, 152.86, 132.96, 132.75, 132.53, 132.32, 131.15, 129.77, 129.61, 128.30, 127.25, 127.00, 126.09, 126.06, 124.77, 122.97, 120.68, 116.56, 113.70, 77.37, 77.16, 76.94, 61.79, 55.65, 55.63, 55.59, 44.30, 10.95, 10.93, 3.70. HRMS *m*/*z* calculated for C_22_H_19_F_3_N_2_O_3_ [M + H]^+^: 417.1420; found: 147.1381.2.3. Cells and Viruses

LLC-MK2 (rhesus monkey kidney epithelial; ATCC CCL-7), Vero E6 (African green monkey kidney epithelial; Vero C1008; KCTC AC28803), and MRC-5 (human lung fibroblast; KCLB 10171) cells were cultured in Dulbecco’s modified Eagle’s medium (DMEM; Corning 10-013-CV, Corning, NY, USA) supplemented with 5% heat-inactivated fetal bovine serum (FBS; Corning 35-016-CV), 1% penicillin–streptomycin (100×; Corning 30-002-CI; 100 U/mL and 100 μg/mL final), and 2 mM L-glutamine (Corning 25-005-CI; 200 mM stock) at 37 °C in a humidified 5% CO_2_ incubator. By exception, MRC-5 cells were maintained in DMEM containing 10% heat-inactivated FBS under otherwise identical conditions. Cells were passaged at ≤80–90% confluence using 0.05% trypsin–EDTA (1×; Corning 25-051-CI) and were routinely screened and found mycoplasma-negative (PCR-based assay).

HCoV-OC43 (KBPV-VR-8), HCoV-NL63 (KBPV-VR-88), and PEDV DR13 (originally described in [20]) were used. HCoV-NL63 was propagated in LLC-MK2 cells, and HCoV-OC43 and PEDV DR13 were propagated in Vero E6 cells. Virus-containing supernatants were clarified (2000× *g*, 10 min, 4 °C), aliquoted, and stored at −80 °C. Infectious titers (TCID_50_/mL) were determined on the corresponding host cell lines by the Spearman–Kärber method with back-titrations included in each experiment [21].

### 2.3. Cytotoxicity (WST) and Antiviral Cytoprotection

Cells were seeded at 2 × 10^4^ cells per well in 96-well plates and, after overnight attachment, exposed to test compounds for 72 h in maintenance medium. The final DMSO concentration was ≤0.1% (*v*/*v*) for all conditions, including vehicle controls. Cell viability was quantified using EZ-Cytox WST reagent (DoGenBio, Seoul, Republic of Korea) according to the manufacturer’s instructions; absorbance was read at 450 nm on a SpectraMax i3x microplate reader (Molecular Devices, San Jose, CA, USA). Viability for each well was normalized to the mean of vehicle-treated (mock) controls on the same plate and expressed as percent viability. Each condition included technical replicates (*n* = 3) per experiment. Concentration–response data from ≥3 independent experiments were fit to a four-parameter logistic (4PL) model in GraphPad Prism 10.1.2 (GraphPad Software, San Diego, CA, USA) (log10-transformed concentrations; bottom and top unconstrained).

For antiviral activity, cells in 96-well plates were infected and treated with test compounds as indicated and incubated for 48–72 h. Cell viability was quantified by WST (EZ-Cytox) at 450 nm on the same reader. Cytoprotective effect was calculated by normalizing to infected DMSO controls and converting to percent protection: % Protection = ((V_drug + virus − V_virus-only)/(V_mock − V_virus-only)) × 100.

### 2.4. Viral RNA Quantification

At 72 h post-infection, 100–200 μL of culture supernatant was collected. Viral RNA was extracted using the QIAamp Viral RNA Mini Kit (Qiagen, Hilden, Germany)according to the manufacturer’s instructions and quantified by one-step RT-qPCR on a real-time thermocycler. Primer/probe sets targeting conserved genomic regions of each virus are listed in Table S2.

EC_50_ values were determined from concentration–response curves generated by 2-fold serial dilutions, with at least four concentration points per curve. Each measurement was performed in triplicate technical replicates. Unless otherwise indicated, data are presented as mean ± SEM from three independent biological experiments (*n* = 3).

For absolute quantification, synthetic DNA (oligonucleotide) standards corresponding to the NL63, OC43, and PEDV amplicons were used. Ten-fold serial dilutions were prepared to generate standard curves. Ct values were interpolated against the curves to obtain gene copies per reaction and converted to genome copies per mL by accounting for extraction and elution volumes and the qPCR input volume.

### 2.5. Immunofluorescence Assay (IFA)

At 48 h post-infection, cells were fixed in 4% paraformaldehyde for 15 min, permeabilized with 0.1% Triton X-100, and blocked with 5% BSA. Samples were incubated with a virus-specific rabbit anti-S protein primary antibody, followed by a goat anti-rabbit IgG H&L (FITC) secondary antibody (Supplementary Table S1) and Hoechst nuclear counterstain. Images were acquired on an epifluorescence microscope. Using Fiji, binary thresholding and particle analysis (or Cell Counter) were applied to count the total number of cells and the number of S-positive cells per field, and the infection rate per well was calculated as (S-positive cells/total cells × 100%). At least eight randomly selected fields per well were analyzed.

### 2.6. Time-of-Addition (TOA) Regimens

TOA experiments were performed in HCoV-NL63–infected LLC-MK2 cells using four regimens:

- Full: compound present from −24 h to 48 h relative to infection.
- Pre-treatment: −24 h to −1 h, wash at −1 h, then infection in drug-free medium.
- Co-treatment: −1 h to 0 h during adsorption only, wash at 0 h.
- Post-treatment: 0 h to 48 h after adsorption.

Unless otherwise specified, infections were carried out at 100 TCID_50_ per well with 1 h adsorption at 37 °C, and compounds were used at 20 µM. Primary readout was S protein IFA at 48 h; EZ-Cytox and TCID_50_ at 48 h for the same regimens are provided in Supplementary Data (Figures S14 and S15).

### 2.7. Host-Response Gene Expression Analysis (Two-Step RT-qPCR)

Total RNA was isolated using the RNeasy kit (QIAGEN, Hilden, Germany) according to the manufacturer’s instructions. cDNA was synthesized with the Tetro cDNA Synthesis Kit (Meridian Bioscience, Memphis, TN, USA) following the manufacturer’s protocol. Quantitative PCR was performed using Luna Universal qPCR Master Mix, SYBR (New England Biolabs, Ipswich, MA, USA). Target genes included IL6, ISG15, IFNB1, NQO1, HMOX1 (HO-1), GCLM, DDIT3 (CHOP), ATF4, and XBP1; primer sequences are listed in Table S3. ACTB served as the internal control. Expression changes were calculated as −ΔΔCt (log_2_ fold change) relative to time-matched calibrators (OC43 only for infected comparisons; DMSO for uninfected). Each condition included *n* = 3 biological replicates. At each time point, data were analyzed independently in GraphPad Prism 10.1.2 (GraphPad Software, San Diego, CA, USA) using an ordinary two-way ANOVA with factors gene (9 levels) and treatment (2 levels). Significance marks reflect within-gene simple-effects comparisons (treatment) with Tukey’s multiple-comparisons adjustment (two-tailed, α = 0.05). Time was not included as a factor; no inferences were made across time points.

### 2.8. Combination (Drug–Drug Interaction) Studies

Concentration matrices were generated within the non-cytotoxic range for KB-2777 + nirmatrelvir (HCoV-NL63/LLC-MK2) and KB-2777 + GS-441524 (HCoV-OC43/Vero E6). Antiviral activity was measured at 48 h post-infection. Interaction surfaces and ZIP synergy scores were computed using SynergyFinder (v3) with default normalization (virus + DMSO control = 0% inhibition). Heatmaps and δ-surfaces were exported directly from SynergyFinder.

### 2.9. Propidium Iodide (PI) Live/Dead Imaging in HCoV-NL63/LLC-MK2 (48 h Post-Infection)

Cells were infected as in the immunofluorescence assay (IFA) and were post-treated with KB-2777 (20 µM, DMSO ≤ 0.1%). At 48 h post-infection, cells were stained live with PI (approximately 1 µg/mL, 30 min) and were immediately imaged (PI/Hoechst). For each field, we counted total nuclei (Hoechst), NL63 S-positive cells, PI-positive cells, and double-positive (S∩PI) cells. Outcomes were % PI^+^/Total and % PI^+^ among infected cells (PI^+^/NL63 S^+^). Bars showed mean ± SEM for *n* = 8 fields per group. Statistics used the Mann–Whitney U test (two-tailed).

### 2.10. Data Processing and Statistics

Curve fitting and EC_50_/CC_50_ calculations were performed in GraphPad Prism 10.1.2 (GraphPad Software, San Diego, CA, USA). Data are presented as mean ± SEM unless indicated. For multi-group comparisons (e.g., TOA IFA, CPE protection, TCID_50_ across regimens), one-way ANOVA with Dunnett’s post hoc test versus the virus control (+DMSO) was used. Exact n values are reported in figure legends. Significance thresholds: *p* < 0.05 (*), 0.01 (**), 0.001 (***), 0.0001 (****). All experiments were repeated in ≥3 independent biological replicates, except where noted (time-course RT-qPCR, *n* = 3 per time).

## 3. Results

### 3.1. Primary Cytotoxicity and Antiviral Screening of Oxazole-4-Carboxamide Derivatives in HCoV-NL63–Infected LLC-MK2 Cells

**Figure 1:**
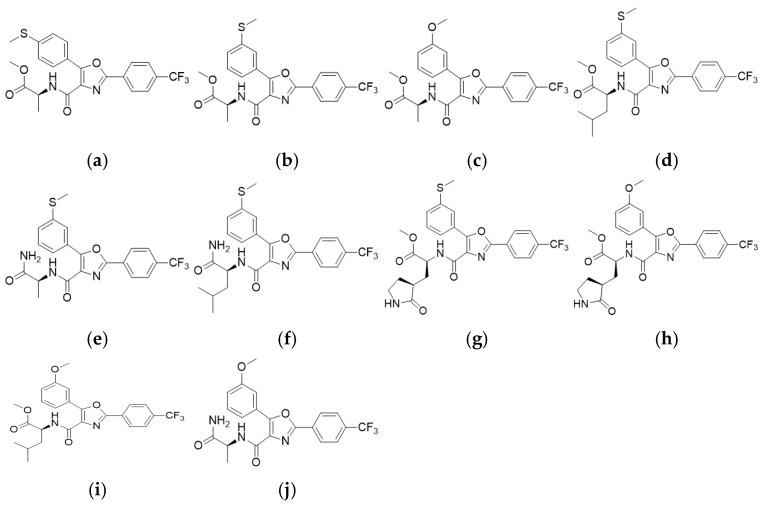
Chemical structures of the test compounds. (**a**) KB-2738 (**b**) KB-2742 (**c**) KB-2767 (**d**) KB-2768 (**e**) KB-2769 (**f**) KB-2771 (**g**) KB-2772 (**h**) KB-2773 (**i**) KB-2774 (**j**) KB-2775.

**Figure 2:**
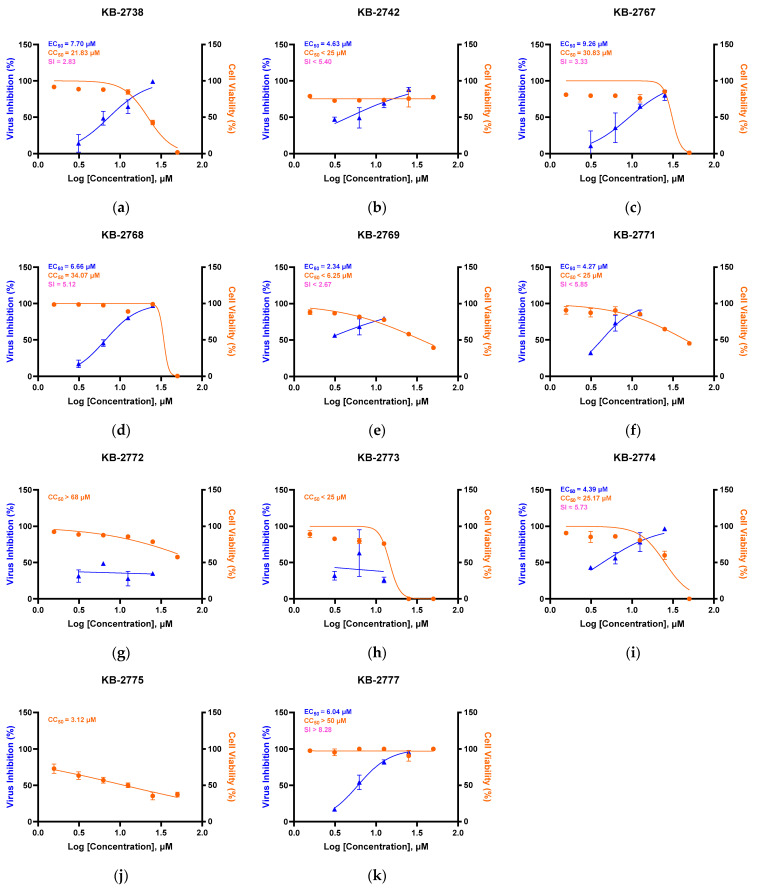
Antiviral and cytotoxic profiles of 11 oxazole-4-carboxamide derivatives. Dose–response curves showing virus inhibition (%) (blue) and cell viability (%) (orange) for compounds KB-2738 (**a**), KB-2742 (**b**), KB-2767 (**c**), KB-2768 (**d**), KB-2769 (**e**), KB-2771 (**f**), KB-2772 (**g**), KB-2773 (**h**), KB-2774 (**i**), KB-2775 (**j**), KB-2777 (**k**). EC_50_, CC_50_, and SI values are indicated in each panel.

We screened 11 oxazole-4-carboxamide derivatives (Figure 1) against HCoV-NL63 in LLC-MK2 cells and quantified antiviral activity and cytotoxicity (Figure 2). Several derivatives inhibited NL63 with distinct selectivity profiles. KB-2738, KB-2742, KB-2767, KB-2768, and KB-2769 yielded EC_50_ = 2.34–9.26 µM with modest selectivity index (SI) values of 2.84–5.12; KB-2769 was further limited by CC_50_ < 6.25 µM. KB-2771 and KB-2774 showed partial inhibition (EC_50_ ≈ 4–5 µM) with low SI. KB-2772, KB-2773, and KB-2775 showed no detectable antiviral effect up to 25 µM; KB-2775 was cytotoxic (CC_50_ = 3.12 µM) (Table 1). We advanced KB-2777 for follow-up based on a balanced profile within the soluble range (EC_50_ = 6.04 µM; CC_50_ > 50 µM; SI > 8). Visible crystals or precipitates formed at ≥50 µM without loss of viability or morphological change; therefore, downstream assays were performed at ≤25 µM.

### 3.2. Antiviral Activity Across α/β-Coronaviruses by RT-qPCR

**Figure 3:**
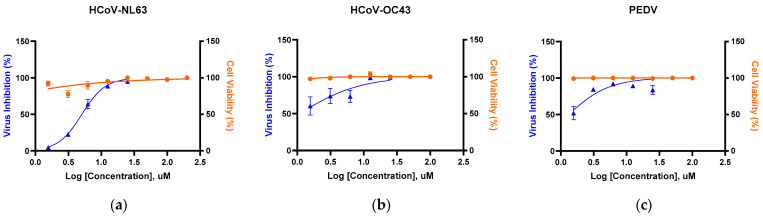
RT-qPCR–based antiviral activity of KB-2777 against representative α/β-coronaviruses (72 h post-infection). Curves show % viral inhibition (blue triangles; RT-qPCR of supernatants) and % cell viability (orange circles; EZ-Cytox in mock-infected cells) under the same dosing. (**a**) HCoV-NL63/LLC-MK2 (EC_50_ = 5.27 μM); (**b**) HCoV-OC43/Vero E6 (EC_50_ = 1.83 μM); (**c**) PEDV/Vero E6 (EC_50_ = 1.59 μM). Data are mean ± SEM (*n*= 3).

We advanced KB-2777, the series member with the highest selectivity index (SI), to evaluate antiviral activity against α-coronaviruses (HCoV-NL63, PEDV) and a β-coronavirus (HCoV-OC43) by RT-qPCR. In all models, KB-2777 reduced viral RNA in a concentration-dependent manner; EC_50_ values were 5.27 µM (HCoV-NL63/LLC-MK2), 1.83 µM (HCoV-OC43/Vero E6), and 1.59 µM (PEDV/Vero E6) (Figure 3). In parallel, EZ-Cytox WST in mock-infected cells showed no overt cytotoxicity across the tested concentration range. Representative cytopathic effect (CPE) micrographs (KB-2777 20 µM vs. virus-only) for HCoV-NL63, HCoV-OC43, and PEDV are provided in the Supplementary Information (Figure S16).

### 3.3. Time-Dependent Antiviral Activity Against HCoV-NL63 (RT-qPCR of Supernatants)

**Figure 4:**
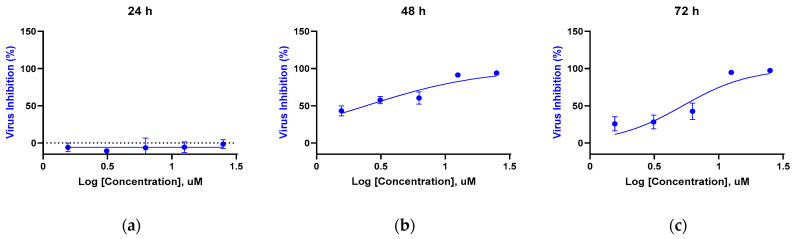
Time-dependent RT-qPCR inhibition by KB-2777 against HCoV-NL63 under the post-treatment regimen. (**a**) 24 h (EC_50_ not determined); (**b**) 48 h (EC_50_ = 2.42 μM); (**c**) 72 h (EC_50_ = 5.25 μM). Points represent mean ± SEM; *n* = 3 (triplicate wells per concentration). The dashed horizontal line in panel (**a**) indicates 0% inhibition.

Time-course experiments were conducted under the post-treatment regimen (see Methods). Viral RNA in culture supernatants was quantified by RT-qPCR at 24, 48, and 72 h post-infection (hpi) across a two-fold dilution series (0–25 μM) (Figure 4). Inhibition at 24 h was minimal (EC_50_ not determined). At 48 h, EC_50_ = 2.42 μM; at 72 h, EC_50_ = 5.25 μM. A modest 48 → 72 h right-shift is expected in multi-cycle assays run without re-dosing, where free drug can decline over prolonged incubations and the 72 h RT-qPCR readout reflects cumulative extracellular viral RNA. 

### 3.4. Infectious Titer Measurement at 48 h (TCID_50_)

**Figure 5:**
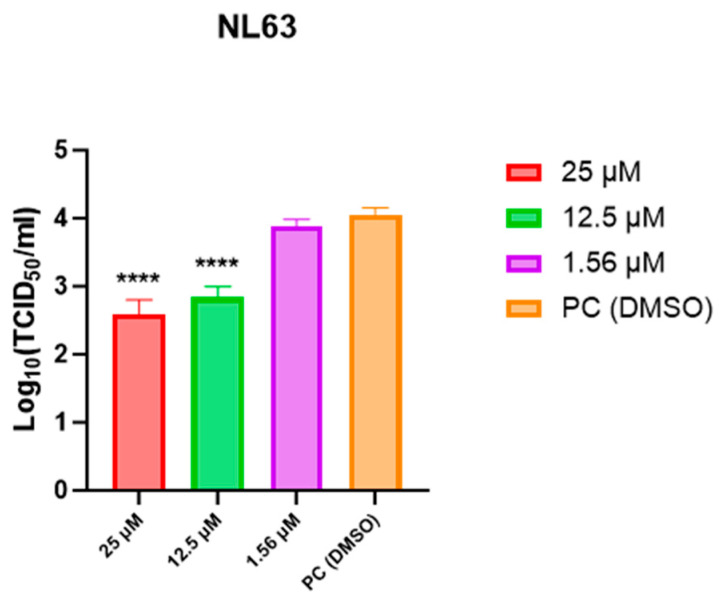
Infectious titer measurement (TCID_50_) of HCoV-NL63 at 48 h after post-treatment with KB-2777 (0, 1.56, 12.5, 25 µM). Infectious titers decreased dose-dependently, with significant reductions at 12.5 µM and 25 µM (**** *p* < 0.0001) relative to the virus control. Bars represent mean ± SEM from three independent experiments (triplicate wells per concentration).

We sought to confirm whether the antiviral activity observed by RT-qPCR was also present at the level of infectious progeny virus. Based on the time-course results, we selected 48 h post-infection, which showed the most pronounced antiviral effect, and conducted infectious titer assays (TCID_50_) using representative concentrations (0, 1.56, 12.5, and 25 µM) corresponding to the effective range determined by RT-qPCR. Infectious titers decreased dose-dependently, showing significant reductions at 12.5 µM and 25 µM compared with the virus control (**** *p* < 0.0001) (Figure 5). Bars represent mean ± SEM; *n* = 3 independent experiments (triplicate wells per concentration).

### 3.5. Time-of-Addition Profile of KB-2777 Against HCoV-NL63 and HCoV-OC43

Time-of-addition (TOA) studies in HCoV-NL63-infected LLC-MK2 cells evaluated four regimens defined in Methods: full (before, during, and after infection), pre-treatment (pre-exposure with washout at inoculation), co-treatment (adsorption only, washout at inoculation), and post-treatment (after adsorption). The primary readout was spike (S) protein immunofluorescence assay (IFA) at 48 h post-infection.

**Figure 6:**
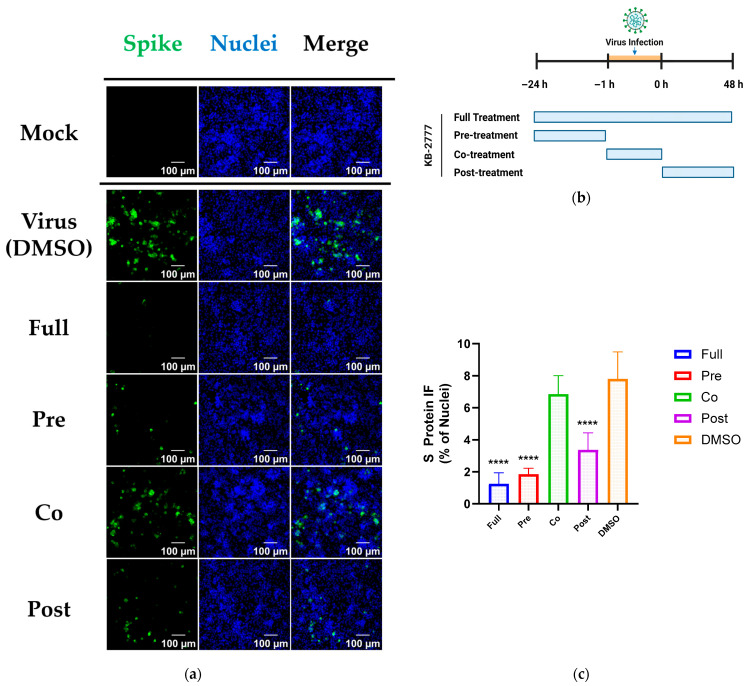
Time-of-addition (TOA) analysis of KB-2777 against HCoV-NL63 (48 h). (**a**) Representative IFA images for each regimen (Spike, green; Nuclei (Hoechst), blue). Acquisition and processing were identical across conditions. Order: Mock, Virus (+DMSO), Full, Pre, Co, Post. Scale bars, 100 μm. (**b**) Schematic of TOA timing. NL63 adsorption(orange shaded): −1 to 0 h. Full: −24 to 48 h; Pre: −24 to 0 h (wash at 0 h); Co: −1 to 0 h only (wash at 0 h); Post: 0 to 48 h. (**c**) Quantified S protein signal expressed as % of virus control (+DMSO = 100%). Bars show mean ± SEM; n ≥ 3; statistics by one-way ANOVA/Dunnett vs. virus control; significance levels shown (**** *p* < 0.0001).

KB-2777 showed the greatest inhibition under the full regimen. Pre- and post-treatment retained measurable activity; co-treatment had little effect relative to the virus-only DMSO control. Infection rate, quantified as the number of S-positive cells normalized to total nuclei, ranked full < pre ≈ post < co. Co-treatment did not differ from the virus-only control (one-way ANOVA with Dunnett’s test; not significant). Representative micrographs and the dosing timeline are shown in Figure 6a,b.

Orthogonal assays for NL63 were concordant with the IFA results. At 48 h post-infection, CPE-based cell protection (EZ-Cytox) at 5 µM and 20 µM reproduced this pattern; endpoint-dilution infectious titers (log_10_[TCID_50_/mL], 50% tissue culture infectious dose) decreased in a regimen-dependent manner, largest with full exposure, smaller with pre- and post-treatment, and minimal with co-treatment and DMSO (Supplementary Figure S14).

For HCoV-OC43, TOA was assessed by CPE reduction in MRC-5 cells and by TCID_50_ on Vero E6 (no IFA collected). Both readouts showed the greatest antiviral effect with full exposure, intermediate activity with pre- and post-treatment, and little or no effect with co-treatment; co-treatment did not differ significantly from DMSO (Supplementary Figure S15).

Collectively, this TOA pattern argued against a purely virucidal or attachment-only mechanism and was consistent with a host-directed and/or early post-entry replication-stage mechanism of action for KB-2777.

### 3.6. Targeted Host-Response Transcript Profiling

Building on antiviral activity confirmed for PEDV, HCoV-NL63, and HCoV-OC43, we profiled host responses across three predefined axes (innate immunity/interferon, NRF2–antioxidant, ER stress/UPR) and quantified time-resolved transcript (mRNA) changes as −ΔΔCt (≈log_2_ fold change) relative to time-matched controls at each time point (OC43-only for infected; DMSO for uninfected; Figure 6). This design isolated treatment effects within each time point. In OC43 alone, IL6 transcripts increased at 24–48 h, ISG15 and XBP1 transcripts rose at later time points, and GCLM transcripts increased gradually. These patterns comprised IL6 mRNA induction, ISG responses, and activation of UPR markers (IRE1α–XBP1, DDIT3), consistent with prior reports [22,23,24]. Fluctuations in NRF2-linked marker transcripts (HMOX1/HO-1, NQO1) aligned with reports linking coronavirus-induced oxidative stress to the NRF2 axis [25,26,27].

KB-2777 co-treatment attenuated these transcriptional responses, with significant decreases in IL6 and IFNB1 mRNA (multiple/mid time points, respectively), ISG15, and selected NRF2/UPR transcripts (NQO1, HMOX1, DDIT3, XBP1) (asterisks in Figure 6). Some markers, notably IL6, remained above the time-matched baseline but were lower than OC43 alone, indicating mitigation rather than complete reversal. Thus, under infection, KB-2777 selectively reduced over-induced inflammatory, IFN/ISG, and UPR signals.

In the KB-2777-only condition, a broad downward shift at 6 h was followed by partial normalization at 12–24 h and selective re-patterning at 48 h (e.g., decreases in ISG15, IFNB1, and DDIT3; asterisks). Changes in IL6, NQO1, HMOX1, XBP1, and ATF4 were small or not significant.

**Figure 7:**
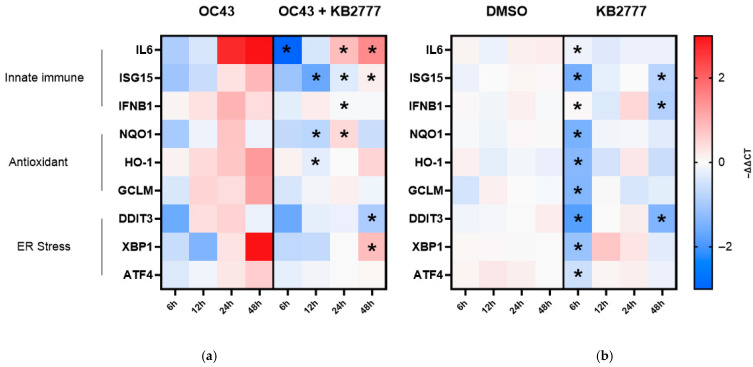
Time-resolved host transcriptional responses to KB-2777 in MRC-5 cells. (**a**) OC43 infection alone (OC43) versus OC43 plus KB-2777 (OC43 + KB-2777). (**b**) Vehicle (DMSO) versus KB-2777 alone under uninfected conditions. Heatmaps display −ΔΔCt (log_2_ fold change). The color scale is centered at 0 in both panels (blue = decrease, red = increase). Row annotations denote functional groups: innate immune (IL6, ISG15, IFNB1), antioxidant/NRF2 (NQO1, HMOX1 [HO-1], GCLM), and ER stress/UPR (DDIT3 [CHOP], XBP1 (total), ATF4). Asterisks indicate Tukey-adjusted *p* < 0.05 from an ordinary two-way ANOVA (factors: gene × treatment) with within-gene comparisons (OC43 vs. OC43 + KB-2777; DMSO vs. KB-2777) performed separately at each time point. * Tukey-adjusted *p* < 0.05 by ordinary two-way ANOVA (gene × treatment); within-gene treatment effect tested at each time point.

Taken together, in infected cells, KB-2777 attenuated hyper-induction along interleukin-6 (IL-6), interferon and interferon-stimulated gene (IFN/ISG; IFNB1, ISG15), and unfolded protein response (UPR; XBP1 total, DDIT3) axes, with modest effects on NRF2 markers. This pattern was consistent with mitigation of stress-amplified host responses rather than broad immunosuppression (Figure 7a). In uninfected cells, changes were limited and transient (Figure 7b). Together with the time-of-addition (TOA) profile reported in Section 3.5 (effect-size order: full > pre ≈ post > co), these data were compatible with early post-entry, endoplasmic reticulum (ER) stress-linked host modulation.

### 3.7. Combination Activity of KB-2777 with Approved DAAs

**Figure 8:**
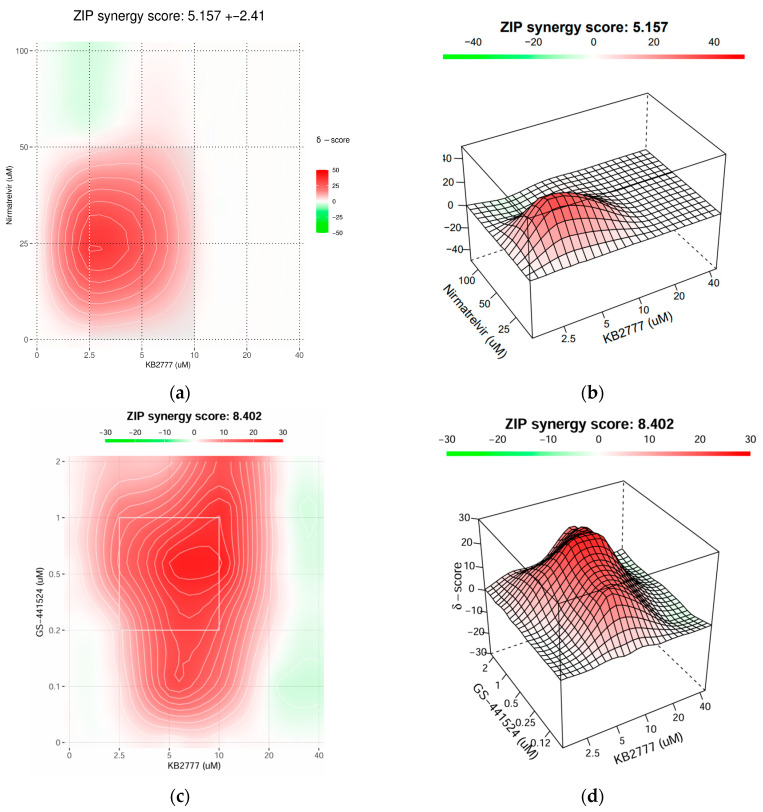
Combination antiviral activity of KB-2777 with direct-acting antivirals. (**a**,**b**) HCoV-NL63/LLC-MK2: concentration–matrix heatmap (**a**) and 3D δ-surface (**b**) for KB-2777 plus nirmatrelvir (3CLpro inhibitor) at 48 h post-infection; ZIP interaction score = 5.157 ± 2.41. (**c**,**d**) HCoV-OC43/Vero E6: heatmap (**c**) and 3D δ-surface (**d**) for KB-2777 plus GS-441524 (RdRp-targeting nucleoside) at 48 h post-infection; ZIP interaction score = 8.402. All combinations were tested within non-cytotoxic/soluble ranges, and responses were normalized to the virus + DMSO control. Corresponding viability measurements are provided in the Supplementary Data.

We tested KB-2777 in combination with nirmatrelvir in the HCoV-NL63/LLC-MK2 model and with GS-441524 in the HCoV-OC43/Vero E6 model using concentration matrix designs and measured antiviral activity at 48 h post-infection (Figure 8).

In the NL63–nirmatrelvir matrix (Figure 8a,b), interactions were additive to mildly synergistic, with a broad synergistic plateau at intermediate concentrations; no pronounced antagonistic ridge was detected, aside from small, localized negative-δ patches. In the OC43–GS-441524 matrix (Figure 8c,d), the synergy region was stronger and wider, yielding a contiguous zone of positive δ values across a larger portion of the surface.

Collectively, KB-2777 combined favorably with DAAs targeting viral protease and polymerase pathways, supporting potential add-on or dose-sparing strategies. The interaction profile was consistent with a host-modulating activity of KB-2777 that complemented DAA mechanisms.

## 4. Discussion

Human coronaviruses (HCoVs) include globally endemic lineages (e.g., OC43, NL63) that cause ~15–30% of common colds, as well as episodically emerging, pandemic-causing betacoronaviruses; recurrent emergence underscores the need for durable antiviral strategies [28,29].

On this basis, we selected oxazole-4-carboxamides as a tractable starting point. The oxazole ring is a privileged heterocycle with broad medicinal chemistry utility and is reported in antiviral and anti-inflammatory chemotypes [13,14]. Heteroaryl-carboxamide motifs also recur across approved and clinical-stage antivirals, supporting compatibility with antiviral design [14]. Guided by this rationale, we synthesized a focused oxazole-4-carboxamide series and identified KB-2777 as the lead candidate. KB-2777 inhibited HCoV-NL63 and showed cross-activity against HCoV-OC43 and PEDV within non-cytotoxic, soluble concentration ranges.

In time-of-addition (TOA) analyses, the rank order across orthogonal readouts was full » pre ≈ post » co, which argues against a purely virucidal or attachment-only mechanism and points to an early post-entry replication stage and/or induction of an antiviral cell state. This assignment aligns with coronavirus biology, in which replication–transcription complexes remodel endoplasmic reticulum (ER) membranes to form double-membrane vesicles (DMVs) and a replication organelle; perturbation of host stress and membrane-organization pathways during this window can affect viral RNA synthesis [30].

Targeted host-response transcript (mRNA) profiling showed context-dependent modulation. In OC43-infected cells, KB-2777 attenuated hyper-induction across inflammatory cytokine and IFN–ISG axes and partially reduced NRF2 marker transcripts (NQO1, HMOX1/HO-1) and selective UPR transcripts (XBP1, DDIT3). In uninfected cells, we observed an early transcriptional decrease at 6 h, partial normalization at 12–24 h, and selective changes at 48 h without a DDIT3 surge, suggesting that a nonspecific stress–UPR-driven effect was less likely. This temporal profile, characterized by adjustment of transcriptional tone without toxicity, accords with reported regulation of NRF2 targets, phased UPR-branch dynamics, and feedback within IFN/ISG pathways. Causal linkage between these transcript-level changes and antiviral activity requires orthogonal validation at the protein and functional levels.

To assess membrane integrity directly, we used propidium iodide (PI) staining in the NL63 system at 48 h post-infection. KB-2777 reduces the percentage of PI-positive cells among total cells and within the infected population. These cytoprotection data align with the TOA signature and with immunofluorescence assay, RT-qPCR, and TCID50 results (Supplementary Figure S17).

From a combination-pharmacology perspective, KB-2777 with standard direct-acting antivirals (DAAs) yielded additive to synergistic activity within non-cytotoxic ranges without antagonism. In the NL63 system, nirmatrelvir alone showed limited activity in our assay window, whereas KB-2777 + nirmatrelvir exceeded either monotherapy. In a betacoronavirus model, KB-2777 + GS-441524 likewise outperformed single agents. Complementary mechanisms can mitigate liabilities of individual target classes and reduce resistance emergence when co-dosed with DAAs, consistent with clinical and evolutionary considerations. Translationally, these combinations may enable dose-sparing, broaden lineage coverage, and increase the genetic barrier to resistance.

In sum, scaffold-guided phenotypic exploration identified KB-2777 as a reproducibly active cell-based lead with cross-model antiviral activity, a TOA signature consistent with host-directed action, and favorable combinability with DAAs. Priority next steps include (i) target deconvolution with orthogonal confirmation; (ii) structure–activity relationship (SAR) optimization and absorption, distribution, metabolism, and excretion (ADME) profiling; (iii) evaluation in primary airway models and in vivo; and (iv) expansion of combinations with clinically relevant antivirals. These directions align with current therapeutic needs and highlight the strategic value of an orthogonal mechanism.

This study uses cell-line models in a single laboratory (LLC-MK2, Vero E6, MRC-5). Validation in primary human airway cultures and in vivo remains necessary. The mechanism of action is unresolved, and host responses are measured at the mRNA level without protein or functional confirmation. These limitations motivate the next steps outlined above.

## Figures and Tables

**Table 1:** EC_50_, CC_50_, and SI of oxazole-4-carboxamide derivatives in HCoV-NL63/LLC-MK2.

Compound	EC_50_ (µM)	CC_50_ (µM)	SI (=CC_50_/EC_50_)
KB-2738	7.70	21.83	2.84
KB-2742	4.63	<25	<5.40
KB-2767	9.26	30.83	3.33
KB-2768	6.66	34.07	5.12
KB-2769	2.34	<6.25	<2.67
KB-2771	4.27	<25	<5.85
KB-2772	-	>68	-
KB-2773	-	<25	-
KB-2774	4.39	25.17	5.73
KB-2775	-	3.12	-
KB-2777	6.04	>50	>8.28

-, not determined.

## Data Availability

The original contributions presented in this study are included in the article/Supplementary Materials. Further inquiries can be directed to the corresponding authors.

## Supplementary Materials

The following supporting information can be downloaded at https://www.mdpi.com/article/10.3390/pharmaceutics17111477/s1, Scheme S1: Synthesis of the KB series. Chemical synthesis. Figures S1–S11: ^1^H NMR spectra of the series members 5a (KB-2738), 5b (KB-2742), 5c (KB-2767), 5d (KB-2768), 5e (KB-2769), 5f (KB-2771), 5g (KB-2772), 5h (KB-2773), 5i (KB-2774), 5j (KB-2775), and 5k (KB-2777). Figure S12: HRMS spectrum of compound 5k (KB-2777). Figure S13: HPLC chromatogram of compound 5k (KB-2777). Figure S14. Time-of-addition (TOA) analysis of KB-2777 against HCoV-NL63 (48 h). Figure S15. Time-of-addition (TOA) analysis of KB-2777 against HCoV-OC43 (48 h). Figure S16. Cytopathic effect (CPE) protection by KB-2777 (20 μM) in coronavirus-infected cells. Figure S17. KB-2777 reduces PI-positive fractions in NL63-infected cultures (48 h post-infection). Table S1: Antibodies used for immunofluorescence. Table S2: Viral RT-qPCR primers/probes. Table S3: Human host-gene primers.

## Abbreviations

The following abbreviations are used in this manuscript:

DAA	Direct-Acting Antiviral
TOA	Time-of-Addition
IFA	Immunofluorescence Assay
RT-qPCR	Reverse Transcription quantitative PCR
CPE	Cytopathic Effect
TCID_50_	50% Tissue Culture Infectious Dose
ER	Endoplasmic Reticulum
UPR	Unfolded Protein Response
NRF2	Nuclear factor erythroid 2–related factor 2
ZIP	Zero-Interaction Potency
HCoV-NL63	Human coronavirus NL63
PEDV	Porcine epidemic diarrhea virus
HCoV-OC43	Human coronavirus OC43
SARS-CoV-2	Severe acute respiratory syndrome coronavirus 2

## References

[1] Chen R., Gao Y., Liu H., Li H., Chen W., Ma J. (2023). Advances in research on 3C-like protease (3CLpro) inhibitors against SARS-CoV-2 since 2020. RSC Med. Chem..

[2] Li X., Song Y. (2023). Structure and function of SARS-CoV and SARS-CoV-2 main proteases and their inhibition: A comprehensive review. Eur. J. Med. Chem..

[3] Iketani S., Mohri H., Culbertson B., Hong S.J., Duan Y., Luck M.I., Annavajhala M.K., Guo Y., Sheng Z., Uhlemann A.-C. (2023). Multiple pathways for SARS-CoV-2 resistance to nirmatrelvir. Nature.

[4] Heilmann E., Costacurta F., Moghadasi S.A., Ye C., Pavan M., Bassani D., Volland A., Ascher C., Weiss A.K.H., Bante D. (2022). SARS-CoV-2 3CLpro mutations selected in a VSV-based system confer resistance to nirmatrelvir, ensitrelvir, and GC376. Sci. Transl. Med..

[5] Zhang L., Xie X., Luo H., Qian R., Yang Y., Yu H., Huang J., Shi P.-Y., Hu Q. (2024). Resistance mechanisms of SARS-CoV-2 3CLpro to the non-covalent inhibitor WU-04. Cell Discov..

[6] Owen D.R., Allerton C.M., Anderson A.S., Aschenbrenner L., Avery M., Berritt S., Boras B., Cardin R.D., Carlo A., Coffman K.J. (2021). An oral SARS-CoV-2 Mpro inhibitor clinical candidate for the treatment of COVID-19. Science.

[7] Gordon C.J., Tchesnokov E.P., Woolner E., Perry J.K., Feng J.Y., Porter D.P., Götte M. (2020). Remdesivir is a direct-acting antiviral that inhibits RNA-dependent RNA polymerase from severe acute respiratory syndrome coronavirus 2 with high potency. J. Biol. Chem..

[8] Choi B., Choudhary M.C., Regan J., Sparks J.A., Padera R.F., Qiu X., Solomon I.H., Kuo H.-H., Boucau J., Bowman K. (2020). Persistence and evolution of SARS-CoV-2 in an immunocompromised host. N. Engl. J. Med..

[9] Baum A., Fulton B.O., Wloga E., Copin R., Pascal K.E., Russo V., Giordano S., Lanza K., Negron N., Ni M. (2020). Antibody cocktail to SARS-CoV-2 spike protein prevents rapid mutational escape seen with individual antibodies. Science.

[10] Greaney A.J., Starr T.N., Barnes C.O., Weisblum Y., Schmidt F., Caskey M., Gaebler C., Cho A., Agudelo M., Finkin S. (2021). Mapping mutations to the SARS-CoV-2 RBD that escape binding by different classes of antibodies. Nat. Commun..

[11] Pawlotsky J.M. (2014). New hepatitis C therapies: The toolbox, strategies, and challenges. Gastroenterology.

[12] Havlir D.V., Tierney C., Friedland G.H., Pollard R.B., Smeaton L., Sommadossi J.-P., Fox L., Kessler H., Fife K.H., Richman D.D. (2000). In vivo antagonism with zidovudine plus stavudine combination therapy. J. Infect. Dis..

[13] Zhang H.-Z., Zhao Z.-L., Zhou C.-H. (2018). Recent advance in oxazole-based medicinal chemistry. Eur. J. Med. Chem..

[14] Patel D., Patel K., Patel S., Patel B., Patel A. (2024). Review on therapeutic diversity of oxazole scaffold: An update. ChemistrySelect.

[15] Meanwell N.A., Belema M. (2019). The discovery and development of daclatasvir: An inhibitor of the hepatitis C virus NS5A replication complex. HCV: The Journey from Discovery to a Cure: Volume II..

[16] Link J.O., Taylor J.G., Xu L., Mitchell M., Guo H., Liu H., Kato D., Kirschberg T., Sun J., Squires N. (2014). Discovery of ledipasvir (GS-5885): A potent, once-daily oral NS5A inhibitor for the treatment of hepatitis C virus infection. J. Med. Chem..

[17] Furuta Y., Komeno T., Nakamura T. (2017). Favipiravir (T-705), a broad spectrum inhibitor of viral RNA polymerase. Proc. Jpn. Acad. Ser. B Phys. Biol. Sci..

[18] Xue F., Luo X., Ye C., Ye W., Wang Y. (2011). Inhibitory properties of 2-substituent-1H-benzimidazole-4-carboxamide derivatives against enteroviruses. Bioorg. Med. Chem..

[19] Kim Y., Ma C., Park S., Shin Y., Lee T., Paek J., Hoon Kim K., Jang G., Cho H., Son S. (2021). Rational Design, Synthesis and Evaluation of Oxazolo[4,5-c]-quinolinone Analogs as Novel Interleukin-33 Inhibitors. Chem. Asian J..

[20] Park S.-J., Song D.-S., Ha G.-W., Park B.-K. (2007). Cloning and further sequence analysis of the spike gene of attenuated porcine epidemic diarrhea virus DR13. Virus Genes.

[21] Kärber G. (1931). Beitrag zur kollektiven Behandlung pharmakologischer Reihenversuche. Naunyn Schmiedebergs Arch. Exp. Pathol. Pharmakol..

[22] Edwards J.A., Denis F., Talbot P.J. (2000). Activation of glial cells by human coronavirus OC43 infection. J. Neuroimmunol..

[23] Liu S.Y., Huang M., Fung T.S., Chen R.A., Liu D.X. (2023). Characterization of the induction kinetics and antiviral functions of IRF1, ISG15 and ISG20 in cells infected with gammacoronavirus avian infectious bronchitis virus. Virology.

[24] Oda J.M., den Hartigh A.B., Jackson S.M., Tronco A.R., Fink S.L. (2023). The unfolded protein response components IRE1α and XBP1 promote human coronavirus infection. mBio.

[25] Qu Y., Haas de Mello A., Morris D.R., Jones-Hall Y.L., Ivanciuc T., Sattler R.A., Paessler S., Menachery V.D., Garofalo R.P., Casola A. (2023). SARS-CoV-2 inhibits NRF2-mediated antioxidant responses in airway epithelial cells and in the lung of a murine model of infection. Microbiol. Spectr..

[26] Gain C., Song S., Angtuaco T., Satta S., Kelesidis T. (2023). The role of oxidative stress in the pathogenesis of infections with coronaviruses. Front. Microbiol..

[27] Daskou M., Fotooh Abadi L., Gain C., Wong M., Sharma E., Kombe Kombe A.J., Nanduri R., Kelesidis T. (2024). The role of the NRF2 pathway in the pathogenesis of viral respiratory infections. Pathogens.

[28] Liu D.X., Liang J.Q., Fung T.S., Bamford D.H., Zuckerman M. (2021). Human Coronavirus-229E, -OC43, -NL63, and -HKU1 (Coronaviridae). Encyclopedia of Virology.

[29] Wang X., Tang G., Liu Y., Zhang L., Chen B., Han Y., Fu Z., Wang L., Hu G., Ma Q. (2022). The role of IL-6 in coronavirus, especially in COVID-19. Front. Pharmacol..

[30] Snijder E.J., Limpens R.W., de Wilde A.H., de Jong A.W., Zevenhoven-Dobbe J.C., Maier H.J., Faas F.F., Koster A.J., Bárcena M. (2020). A unifying structural and functional model of the coronavirus replication organelle: Tracking down RNA synthesis. PLoS Biol..

