# Supplementary material for: Screening-Identified Oxazole-4-Carboxamide KB-2777 Exhibits In Vitro Anti-Coronavirus Activity
Source: Pharmaceutics. 2025 Nov 16;17(11):1477. doi: 10.3390/pharmaceutics17111477 (PMC12655682; doi:10.3390/pharmaceutics17111477)
Supplement: Supplementary file 1 [file pharmaceutics-17-01477-s001.zip › pharmaceutics-3933727-supplementary.pdf]

Supplementary Information

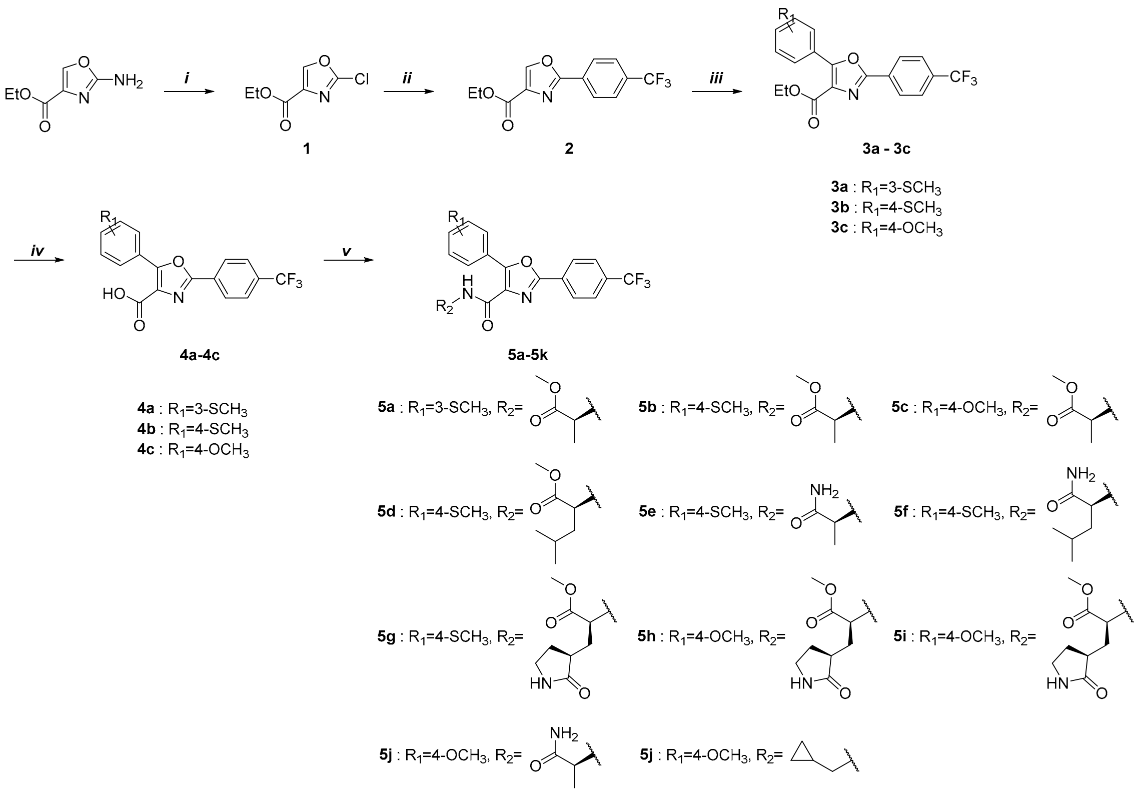

Reagents and conditions: (i) t-BuONO, $CuCl_2$, ACN, 80 °C, 6 h; (ii) 4-(trifluoromethyl)phenylboronic acid, $Pd(PPh_3)_4$, K_2CO_3, Toluene/H_2O, 80 °C, 16 h; (iii) aryl bromide, $Pd(OAc)_2$, $P(o\text{-}tol)_3$, Cs_2CO_3, toluene, 90 °C, 16 h; (iv) 1M NaOH, EtOH, r.t, 6 h; (v)amine, EDC-HCl, HOBt, DMF, r.t, 6 h.

Scheme S1. Synthesis of the KB series

Chemical synthesis.

Ethyl 2-chlorooxazole-4-carboxylate (**1**)

Ethyl 2-aminooxazole-4-carboxylate (5.0 g, 32.0 mmol) was added in portions to a solution of tert-butyl nitrite (1.5 eq) and copper(II) chloride (1.5 eq) in acetonitrile (150 mL). The reaction mixture was stirred under argon at room temperature for 0.5 h and then heated at 80 °C for 6 h. The solution was cooled and partitioned between dichloromethane, water, and concentrated HCl. After cooling to ambient temperature, the mixture was partitioned between dichloromethane, water, and concentrated HCl. The aqueous phase was extracted with dichloromethane, and the combined organic layers were washed with brine, dried over anhydrous $MgSO_4$, and concentrated under reduced pressure. The crude residue was purified by column chromatography on silica gel (hexane/Et2O = 11/1 to 9/1, v/v) to furnish compound **1** (3.09 g, 17.6 mmol, 55%) as a white solid. R_f = 0.43 (hexane/Et2O = 2/1, v/v). ^{1}H NMR (600 MHz, $CDCl_3$) δ 8.19 (s, 1H), 4.40 (q, J = 7.2 Hz, 2H), 1.39 (t, J = 7.2 Hz, 3H).

Ethyl 2-(4-(trifluoromethyl)phenyl)oxazole-4-carboxylate (**2**)

To a solution of compound 1 (358 mg, 2.04 mmol) and 4-(trifluoromethyl)phenylboronic acid (1.5 eq) in toluene was added a solution of K_2CO_3 (2.0 eq) in H_2O, followed by tetrakis(triphenylphosphine)palladium(0) (0.05 eq). The reaction mixture was stirred under argon at 80 °C for 16 h. After cooling to room temperature, the mixture was partitioned between ethyl acetate and water, and the aqueous layer was further extracted with ethyl acetate. The combined organic extracts were washed with 3 N HCl, dried over anhydrous $MgSO_4$, and concentrated under reduced pressure. The crude residue was purified by column chromatography on silica gel (hexane/ethyl acetate

= 9:1 → 6:1, v/v) to afford compound **2** as a white solid (302 mg, 1.06 mmol, 52%). R_f=0.61 (Hexane/Ethyl acetate = 2:1, v/v). ^{1}H NMR (600 MHz, $CDCl_3$) δ 8.35 (s, 1H), 8.25 (d, J = 7.8 Hz, 2H), 7.75 (d, J = 8.4 Hz, 2H), 4.47 (q, J = 7.2 Hz, 2H), 1.44 (t, J = 7.1 Hz, 3H).

General Procedure A for the Preparation of Compounds **3a–3c**

Compound **2** (1.0 eq), an aryl bromide (1.2 eq; 3-bromothioanisole, 4-bromothioanisole, or 3-bromoanisole), cesium carbonate (Cs_2CO_3, 1.5 eq), tri(o-tolyl)phosphine (0.20 eq), and palladium(II) acetate (0.20 eq) were dissolved in toluene (20 mL) and stirred under argon at 90 °C for 16 h. After cooling to room temperature, the reaction mixture was partitioned between ethyl acetate, water, and 3 N HCl. The aqueous layer was extracted with ethyl acetate and the combined organic layers were washed with water and brine, dried over anhydrous $MgSO_4$, filtered, and concentrated under reduced pressure. The crude residue was purified by silica gel column chromatography (hexane/ethyl acetate = 9:1 → 7:1, v/v) to afford the corresponding solids **3a-3c**.

Ethyl 5-(4-(methylthio)phenyl)-2-(4-(trifluoromethyl)phenyl)oxazole-4-carboxylate (**3a**)

Compound **3a** (219 mg, 0.54 mmol) was prepared in 76.0% yield as a white solid, by following the same method as described in the general procedure A with 4-bromothioanisole. R_f = 0.60 (hexane/ethyl acetate = 2/1, v/v). ^{1}H NMR (600 MHz, $CDCl_3$) δ 8.27 (d, J = 8.2 Hz, 2H), 8.07 (d, J = 8.7 Hz, 2H), 7.75 (d, J = 8.2 Hz, 2H), 7.35 (d, J = 8.7 Hz, 2H), 4.47 (q, J = 7.2 Hz, 2H), 2.55 (s, 3H), 1.44 (t, J = 7.2 Hz, 3H).

Ethyl 5-(3-(methylthio)phenyl)-2-(4-(trifluoromethyl)phenyl)oxazole-4-carboxylate (**3b**)

Compound **3b** (614 mg, 1.50 mmol) was prepared in 85.0% yield as a white solid, by following the same method as described in the general procedure A with 3-bromothioanisole. R_f = 0.60 (hexane/ethyl acetate = 2/1, v/v). ^{1}H NMR (600 MHz, $CDCl_3$) δ 8.28 (d, J = 8.7 Hz, 2H), 8.05 (t, J = 1.8 Hz, 1H), 7.86 (dt, J = 7.8, 1.4 Hz, 1H), 7.76 (d, J = 8.4 Hz, 2H), 7.43 (t, J = 7.8 Hz, 1H), 7.38 (dt, J = 7.9, 1.5 Hz, 1H), 4.47 (q, J = 7.1 Hz, 2H), 2.57 (s, 3H), 1.43 (t, J = 7.1 Hz, 3H).

Ethyl 5-(3-methoxyphenyl)-2-(4-(trifluoromethyl)phenyl)oxazole-4-carboxylate (**3c**)

Compound **3c** (154 mg, 0.39 mmol) was prepared in 51.0% yield as a white solid, by following the same method as described in the general procedure A with 3-bromoanisole. R_f = 0.58 (hexane/ethyl acetate = 2:1, v/v). ^{1}H NMR (600 MHz, $CDCl_3$) δ 8.27 (d, J = 8.2 Hz, 2H), 7.75 (app, J = 8.2 Hz, 1H, overlapped with s, 1H), 7.68 (d, J = 7.8 Hz, 1H), 7.42 (dd, J = 7.8,8.2 Hz, 1H), 7.04 (d, J = 8.2 Hz, 1H), 4.47 (q, J = 7.1 Hz, 2H), 3.90 (s, 3H), 1.43 (t, J = 7.1 Hz, 3H).

General Procedure B for the Preparation of Compounds **4a–4c**

Compounds **3a–3c** (1.0 equiv) were dissolved in ethanol (30 mL), and NaOH (1 M, 3.0 equiv) was added. The mixture was stirred under argon at room temperature for 6 h. The solvent was removed under reduced pressure, and the residue was diluted with water and acidified with 3 N HCl to pH 1–2. The precipitate was collected by filtration, washed with water, and dried to afford the corresponding solids **4a–4c**.

5-(4-(methylthio)phenyl)-2-(4-(trifluoromethyl)phenyl)oxazole-4-carboxylic acid (**4a**)

Compound **4a** (150 mg, 0.39 mmol) was prepared in 84.0% yield as a white solid, by following the same method as described in the general procedure B. Rf = 0.60 (hexane/ethyl acetate = 2/1, v/v). ^{1}H NMR (600 MHz, DMSO) δ 8.34 (d, J = 8.5 Hz, 2H), 8.30 (d, J = 8.4 Hz, 2H), 7.91 (d, J = 8.5 Hz, 2H), 7.33 (d, J = 8.7 Hz, 2H), 2.53 (s, 3H).

5-(3-(methylthio)phenyl)-2-(4-(trifluoromethyl)phenyl)oxazole-4-carboxylic acid (**4b**)

Compound **4b** (212 mg, 0.56 mmol) was prepared in 91.0% yield as a white solid, by following the same method as described in the general procedure B. R_f =0.02 (Dichloromethane/MeOH = 10:1, v/v). ^{1}H NMR (600 MHz, DMSO) δ 8.33 (d, J = 8.5 Hz, 2H), 8.10 (t, J = 1.8 Hz, 1H), 7.96 (d, J = 8.2 Hz, 2H), 7.89 (dt, J = 7.8, 1.2 Hz, 1H), 7.51 (t, J = 7.8 Hz, 1H), 7.43 (ddd, J = 8.2, 2.1, 1.2 Hz, 1H), 2.55 (s, 3H).

5-(3-methoxyphenyl)-2-(4-(trifluoromethyl)phenyl)oxazole-4-carboxylic acid (**4c**)

Compound **4c** (120 mg, 0.33 mmol) was prepared in 85.0% yield as a white solid, by following the same method as described in the general procedure B. R_f =0.45 (Dichloromethane/MeOH = 10:1, v/v). ^{1}H NMR (600 MHz, DMSO-d_6) δ 8.32 (d, J = 8.2 Hz, 2H), 7.96 (d, J = 8.2 Hz, 2H), 7.82-7.76 (m, 1H), 7.71 (d, J = 7.9 Hz, 1H), 7.48 (dd, J = 7.9, 8.3 Hz, 1H), 7.13 (d, J = 8.3 Hz, 1H), 3.84 (s, 3H).

General Procedure C for the Preparation of Compounds **5a–5k**

Compounds **4a–4c** (1.0 eq), HOBt (1.6 eq), and EDC·HCl (1.6 eq) were dissolved in DMF under argon at room temperature. An amine (1.2 eq) was then added, and the reaction mixture was stirred at the same temperature until TLC indicated complete consumption of the starting material (typically 6 h). The mixture was diluted with ethyl acetate and water, and the layers were separated. The aqueous layer was extracted with ethyl acetate, and the combined organic layers were washed with brine, dried over anhydrous $MgSO_4$, filtered, and concentrated under reduced pressure. The residue was purified by silica gel column chromatography (hexane/ethyl acetate = 7:1 → 2:1, v/v) to afford the corresponding solids **5a–5k**.

Methyl (5-(4-(methylthio)phenyl)-2-(4-(trifluoromethyl)phenyl)oxazole-4-carbonyl)-D-alaninate (**5a, KB-2738**)

Compound **5a** (114 mg, 0.24 mmol) was obtained as a white solid in 89.0% yield by following general procedure C, using acid 4a and L-alanine methyl ester hydrochloride as the amine component. ^{1}H NMR (600 MHz, $CDCl_3$) δ 8.33 – 8.28 (m, 2H), 8.24 (d, J = 8.4 Hz, 2H), 7.84 (d, J = 7.6 Hz, 1H), 7.77 (d, J = 8.4 Hz, 2H), 7.33 (d, J = 8.7 Hz, 2H), 4.81 (p, J = 7.3 Hz, 1H), 3.81 (s, 3H), 2.53 (s, 3H), 1.58 (d, J = 7.2 Hz, 3H).

Methyl (5-(3-(methylthio)phenyl)-2-(4-(trifluoromethyl)phenyl)oxazole-4-carbonyl)-D-alaninate **(5b, KB-2742)**

Compound **5b** (107 mg, 0.23 mmol) was obtained as a white solid in 39.0% yield by following general procedure C, using acid 4b and L-alanine methyl ester hydrochloride as the amine component. ^{1}H NMR (600 MHz, $CDCl_3$) δ 8.30 (t, J = 1.8 Hz, 1H), 8.27 – 8.23 (m, 2H), 8.14 (ddd, J = 7.8, 1.7, 1.1 Hz, 1H), 7.85 (d, J = 8.1 Hz, 1H), 7.78 (d, J = 8.1 Hz, 2H), 7.40 (d, J = 7.8 Hz, 1H), 7.34 (ddd, J = 7.8, 1.7, 1.1 Hz, 1H), 4.83 (p, J = 7.3 Hz, 1H), 3.81 (s, 3H), 2.57 (s, 3H), 1.58 (d, J = 7.2 Hz, 3H).

Methyl (5-(3-methoxyphenyl)-2-(4-(trifluoromethyl)phenyl)oxazole-4-carbonyl)-D-alaninate **(5c, KB-2767)**

Compound **5c** (67 mg, 0.08 mmol) was obtained as a white solid in 65.0% yield by following general procedure C, using acid 4c and L-alanine methyl ester hydrochloride as the amine component. ^{1}H NMR (600 MHz, $CDCl_3$) δ 8.25 (d, J = 8.1 Hz, 2H), 8.08 (dd, J = 2.6, 1.6 Hz, 1H), 7.92 (dt, J = 7.8, 1.3 Hz, 1H), 7.87 (d, J = 7.8 Hz, 1H), 7.77 (d, J = 8.4 Hz, 2H), 7.41 (t, J = 8.1 Hz, 1H), 7.01 (ddd, J = 8.4, 2.6, 0.9 Hz, 1H), 4.83 (p, J = 7.3 Hz, 1H), 3.91 (s, 3H), 3.81 (s, 3H), 1.58 (d, J = 7.3 Hz, 3H).

Methyl (5-(3-(methylthio)phenyl)-2-(4-(trifluoromethyl)phenyl)oxazole-4-carbonyl)-D-leucinate **(5d, KB-2768)**

Compound **5d** (45 mg, 0.08 mmol) was obtained as a white solid in 32.0% yield by following general procedure C, using acid 4b and L-leucine methyl ester hydrochloride as the amine component. ^{1}H NMR (600 MHz, $CDCl_3$) δ 8.27 – 8.25 (m, 2H), 8.24 (s, 1H), 8.13 (dt, J = 7.8, 1.2 Hz, 1H), 7.78 (d, J = 8.2 Hz, 2H), 7.71 (d, J = 8.5 Hz, 1H), 7.40 (d, J = 7.8 Hz, 1H), 7.35 – 7.32 (m, 1H), 4.90 – 4.80 (m, 1H), 3.78 (s, 3H), 2.57 (s, 3H), 1.83 – 1.72 (m, 3H), 1.03 – 0.95 (m, 6H).

(S)-N-(1-amino-1-oxopropan-2-yl)-5-(3-(methylthio)phenyl)-2-(4-(trifluoromethyl)phenyl)oxazole-4-carboxami

-de **(5e, KB-2769)**

Compound **5e** (60 mg, 0.13 mmol) was obtained as a white solid in 51.0% yield by following general procedure C, using acid 4b and L-alaninamide hydrochloride as the amine component. ^{1}H NMR (600 MHz, $CDCl_3$) δ 8.31 (s, 1H), 8.24 (d, J = 8.2 Hz, 2H), 8.10 (d, J = 7.8 Hz, 1H), 7.78 (d, J = 8.4 Hz, 3H), 7.42 (t, J = 7.8 Hz, 1H), 7.36 (d, J = 8.2 Hz, 1H), 6.25 (s, 1H), 5.39 (s, 1H), 4.73 (t, J = 7.3 Hz, 1H), 2.58 (s, 3H), 1.59 – 1.53 (m, 3H).

(S)-N-(1-amino-4-methyl-1-oxopentan-2-yl)-5-(3-(methylthio)phenyl)-2-(4-(trifluoromethyl)phenyl)Oxazole-4

-carboxamide **(5f, KB-2771)**

Compound **5f** (71 mg, 0.14 mmol) was obtained as a white solid in 56.0% yield by following general procedure C, using acid 4b and L-leucine amide hydrochloride as the amine component. ^{1}H NMR (600 MHz, $CDCl_3$) δ 8.29 (t, *J* = 1.8 Hz, 1H), 8.23 (d, *J* = 8.1 Hz, 2H), 8.11 – 8.09 (m, 1H), 7.78 (d, *J* = 8.4 Hz, 2H), 7.68 (d, *J* = 8.7 Hz, 1H), 7.41 (t, *J* = 7.8 Hz, 1H), 7.35 (ddd, *J* = 7.9, 2.0, 1.2 Hz, 1H), 6.36 (s, 1H), 5.59 – 5.51 (m, 1H), 4.71 (td, *J* = 8.7, 5.7 Hz, 1H), 2.57 (s, 3H), 1.92 – 1.86 (m, 1H), 1.83 – 1.72 (m, 2H), 1.00 (dd, *J* = 13.9, 6.5 Hz, 6H).

Methyl (S)-2-(5-(3-(methylthio)phenyl)-2-(4-(trifluoromethyl)phenyl)oxazole-4-carboxamido)-3-((S)-2-oxopyrrolidin-3-yl)propanoate **(5g, KB-2772)**

Compound **5g (**19 mg, 0.03 mmol) was obtained as a white solid in 12.0% yield by following general procedure C, using acid 4b and L-leucine amide hydrochloride as the amine component. ^{1}H NMR (600 MHz, $CDCl_3$) δ 8.29 (t, *J* = 1.8 Hz, 1H), 8.26 (d, *J* = 8.1 Hz, 2H), 8.12 (dt, *J* = 7.8, 1.5 Hz, 1H), 8.07 (d, *J* = 8.5 Hz, 1H), 7.78 (d, *J* = 8.5 Hz, 2H), 7.41 (t, *J* = 7.8 Hz, 1H), 7.34 (ddd, *J* = 7.8, 1.9, 1.0 Hz, 1H), 5.98 (s, 1H), 4.89 (ddd, *J* = 10.7, 8.7, 3.8 Hz, 1H), 3.80 (s, 3H), 3.40 – 3.28 (m, 2H), 2.53 (ddd, *J* = 8.7, 5.3, 1.7 Hz, 2H), 2.41 (ddd, *J* = 14.5, 10.7, 3.8 Hz, 1H), 2.03 (ddd, *J* = 14.1, 9.7, 3.9 Hz, 1H), 1.96 – 1.88 (m, 1H).

Methyl (S)-2-(5-(3-methoxyphenyl)-2-(4-(trifluoromethyl)phenyl)oxazole-4-carboxamido)-3-((S)-2-oxopyrrolidin-3-yl)propanoate **(5h, KB-2773)**

Compound **5g (**23 mg, 0.04 mmol) was obtained as a white solid in 14.0% yield by following general procedure C, using acid 4c and methyl (S)-2-amino-3-((S)-2-oxopyrrolidin-3-yl)propanoate as the amine component. ^{1}H NMR (600 MHz, $CDCl_3$) δ 8.28 – 8.23 (m, 2H), 8.07 – 8.03 (m, 2H), 7.91 (ddd, *J* = 7.8, 1.7, 1.0 Hz, 1H), 7.78 (d, *J* = 8.1 Hz, 2H), 7.41 (t, *J* = 8.0 Hz, 1H), 7.02 – 6.99 (m, 1H), 5.74 – 5.69 (m, 1H), 4.89 (ddd, *J* = 10.7, 8.7, 3.9 Hz, 1H), 3.90 (s, 3H), 3.79 (s, 3H), 3.39 – 3.29 (m, 2H), 2.56 – 2.48 (m, 2H), 2.41 (ddd, *J* = 14.5, 10.7, 3.9 Hz, 1H), 2.03 (ddd, *J* = 14.1, 9.7, 4.0 Hz, 1H), 1.97 – 1.88 (m, 1H).

Methyl (5-(3-methoxyphenyl)-2-(4-(trifluoromethyl)phenyl)oxazole-4-carbonyl)-D-leucinate **(5i, KB-2774)**

Compound **5g (**23 mg, 0.04 mmol) was obtained as a white solid in 14.0% yield by following general procedure C, using acid 4c and methyl (S)-2-amino-3-((S)-2-oxopyrrolidin-3-yl)propanoate as the amine component. ^{1}H NMR (600 MHz, $CDCl_3$) δ 8.25 (d, *J* = 8.4 Hz, 2H), 8.05 (s, 1H), 7.93 (dd, *J* = 7.8, 1.3 Hz, 1H), 7.77 (d, *J* = 8.4 Hz, 2H), 7.69 (d, *J* = 8.8 Hz, 1H), 7.40 (t, *J* = 8.1 Hz, 1H), 7.00 (dd, *J* = 8.3, 2.7 Hz, 1H), 4.86 (td, *J* = 8.8, 5.0 Hz, 1H), 3.90 (s, 3H), 3.78 (s, 3H), 1.84 – 1.72 (m, 3H), 1.01 (d, *J* = 6.2 Hz, 6H).

(S)-N-(1-amino-1-oxopropan-2-yl)-5-(3-methoxyphenyl)-2-(4-(trifluoromethyl)phenyl)oxazole-4-carboxamide **(5j, KB-2775)**

Compound **5j** (30 mg, 0.06 mmol) was obtained as a white solid in 31.0% yield by following general procedure C, using acid 4c and L-alanine methyl ester hydrochloride as the amine component. ^{1}H NMR (600 MHz, $CDCl_3$) δ 8.24 (d, *J* = 8.1 Hz, 2H), 8.07 (dd, *J* = 2.6, 1.8 Hz, 1H), 7.90 – 7.86 (m, 1H), 7.78 (d, *J* = 8.2 Hz, 3H), 7.42 (t, *J* = 8.1 Hz, 1H), 7.03 (ddd, *J* = 8.2, 2.6, 0.9 Hz, 1H), 6.26 (s, 1H), 5.35 (t, *J* = 4.8 Hz, 1H), 4.73 (p, *J* = 7.1 Hz, 1H), 3.91 (s, 3H), 1.57 (d, *J* = 7.0 Hz, 3H).

N-(cyclopropylmethyl)-5-(3-methoxyphenyl)-2-(4-(trifluoromethyl)phenyl)oxazole-4-carboxamide (**5K, KB-2777**)

Compound **5k** (241 mg, 0.58 mmol) was obtained as a white solid in 86.0% yield by following general procedure C, using acid 4c and cyclopropylmethylamine as the amine component. ^{1}H NMR (600 MHz, $CDCl_3$) δ 8.24 (d, *J* = 8.1 Hz, 2H), 8.18 – 8.13 (m, 1H), 7.93 (d, *J* = 8.3 Hz, 2H), 7.77 (d, *J* = 8.1 Hz, 2H), 7.49 (brs, 1H), 7.40 (dd, *J* = 7.8, 8.0 Hz, 1H), 7.00 (d, *J* = 8.0 Hz, 1H), 3.92 (s, 3H), 3.39 – 3.32 (m, 2H), 1.16 – 1.07 (m, 1H), 0.63 – 0.56 (m, 2H), 0.35 – 0.29 (m, 2H). ^{13}C NMR (151 MHz, $CDCl_3$) *δ* 161.04, 159.71, 157.00, 152.86, 132.96, 132.75, 132.53, 132.32, 131.15, 129.77, 129.61, 128.30, 127.25, 127.00, 126.09, 126.06, 124.77, 122.97, 120.68, 116.56, 113.70, 77.37, 77.16, 76.94, 61.79, 55.65, 55.63, 55.59, 44.30, 10.95, 10.93, 3.70. HRMS m/z calculated for $C_{22}H_{19}F_3N_2O_3$ $[M+H]^+$: 417.1420; found: 147.1381.

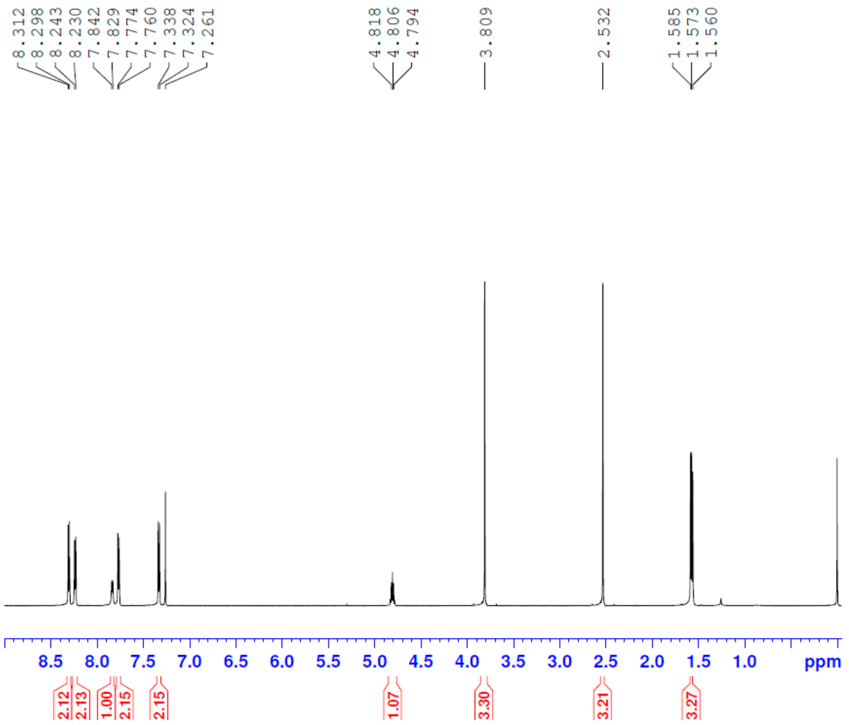

Figure S1. ^{1}H NMR spectra of compound **5a** (KB-2738) measured in $CDCl_3$ at 600 MHz

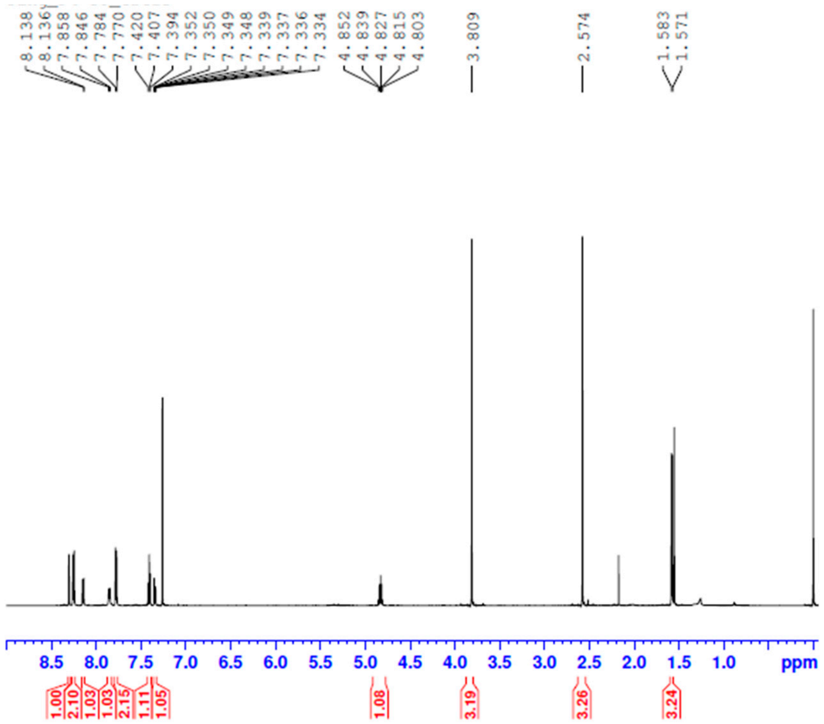

Figure S2. ^{1}H NMR spectra of compound **5b** (KB-2742) measured in $CDCl_3$ at 600 MHz

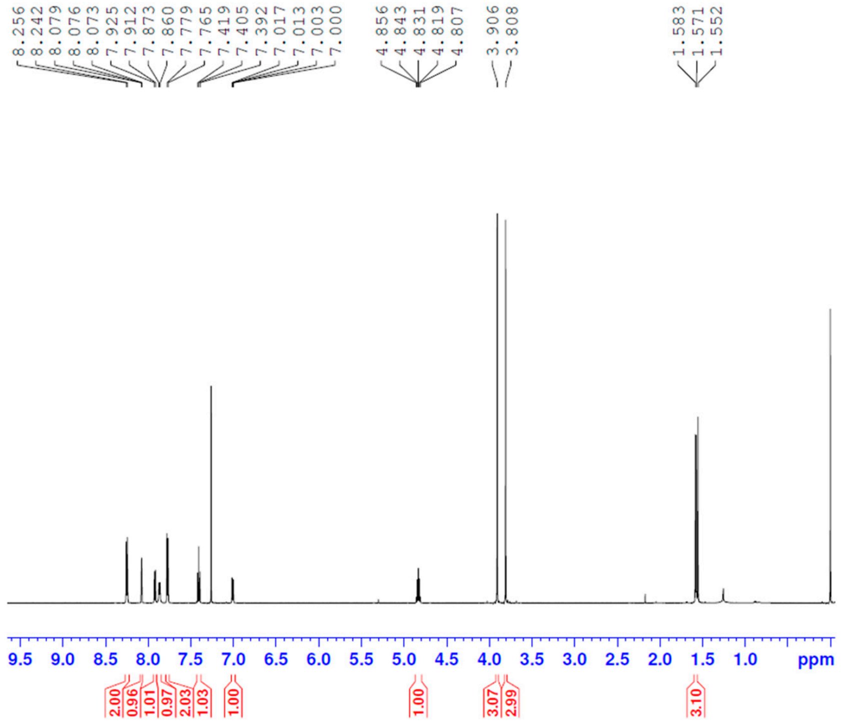

Figure S3. ^{1}H NMR spectra of compound **5c** (KB-2767) measured in $CDCl_3$ at 600 MHz

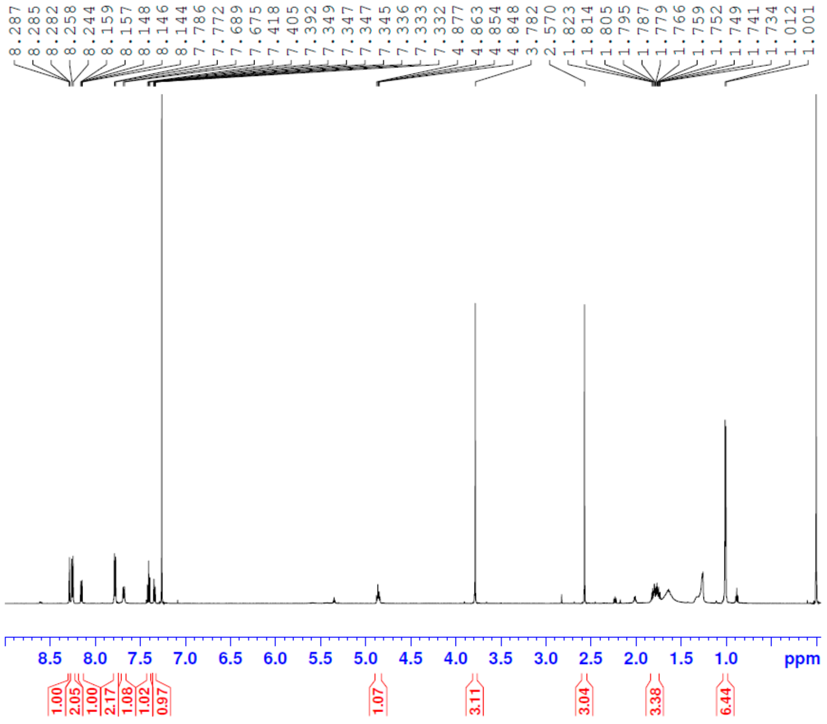

Figure S4. ^{1}H NMR spectra of compound **5d** (KB-2768) measured in $CDCl_3$ at 600 MHz

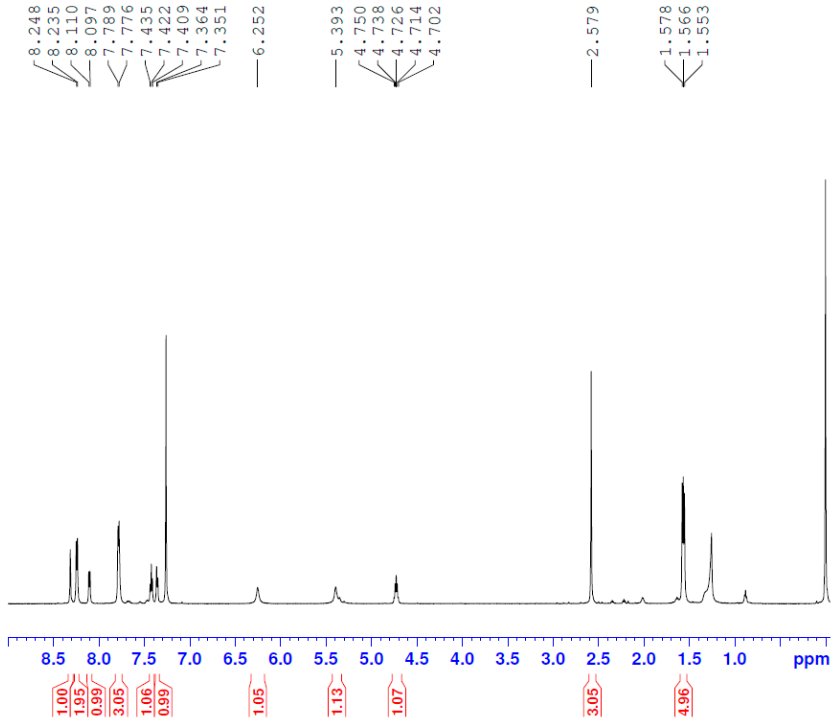

Figure S5. ^{1}H NMR spectra of compound **5e** (KB-2769) measured in $CDCl_3$ at 600 MHz

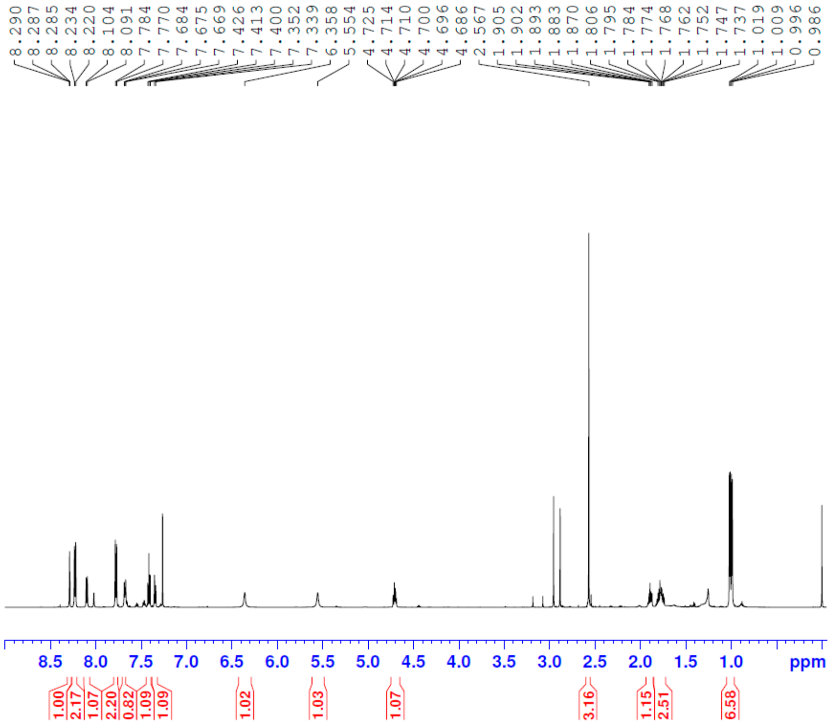

Figure S6. ^{1}H NMR spectra of compound **5f** (KB-2771) measured in $CDCl_3$ at 600 MHz

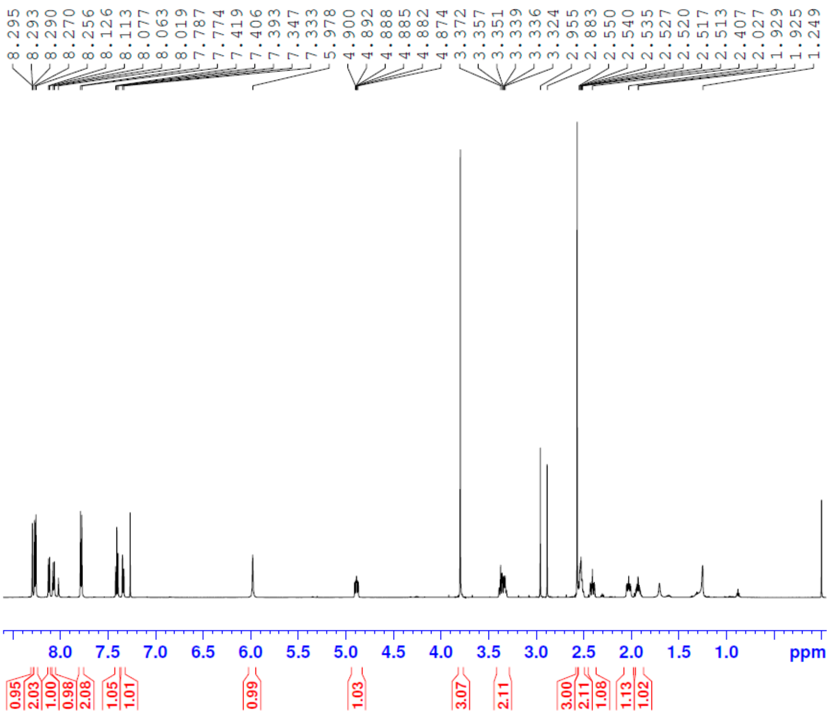

Figure S7. ^{1}H NMR spectra of compound **5g** (KB-2772) measured in $CDCl_3$ at 600 MHz

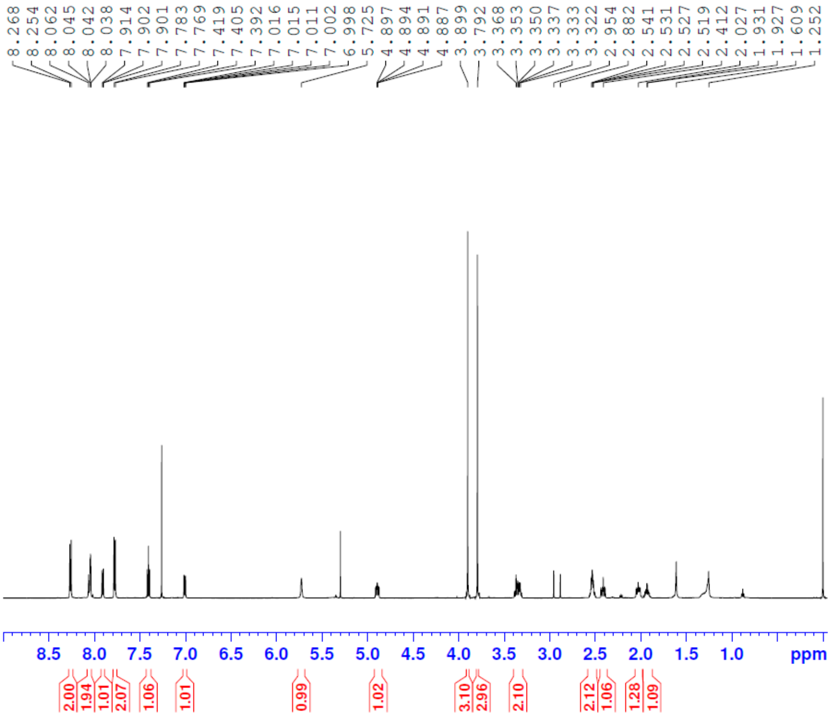

Figure S8. ^{1}H NMR spectra of compound **5h** (KB-2773) measured in $CDCl_3$ at 600 MHz

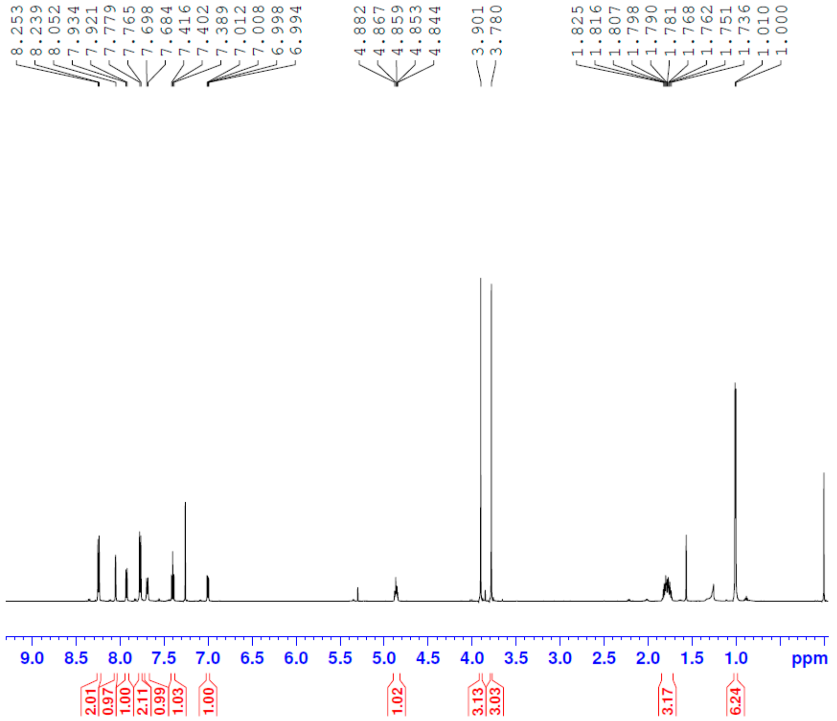

Figure S9. ^{1}H NMR spectra of compound **5i** (KB-2774) measured in $CDCl_3$ at 600 MHz

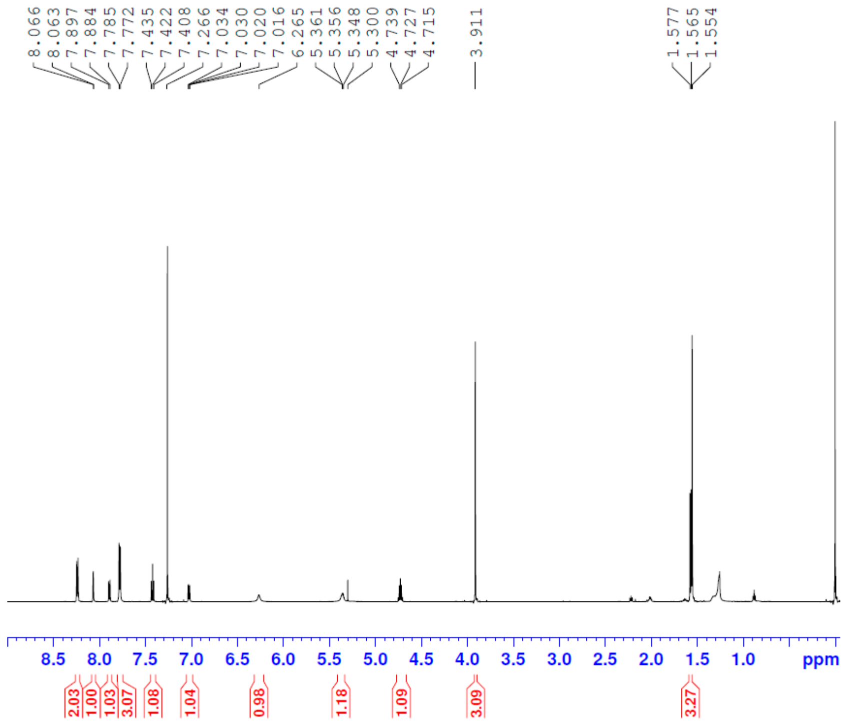

Figure S10. ^{1}H NMR spectra of compound **5j** (KB-2775) measured in $CDCl_3$ at 600 MHz

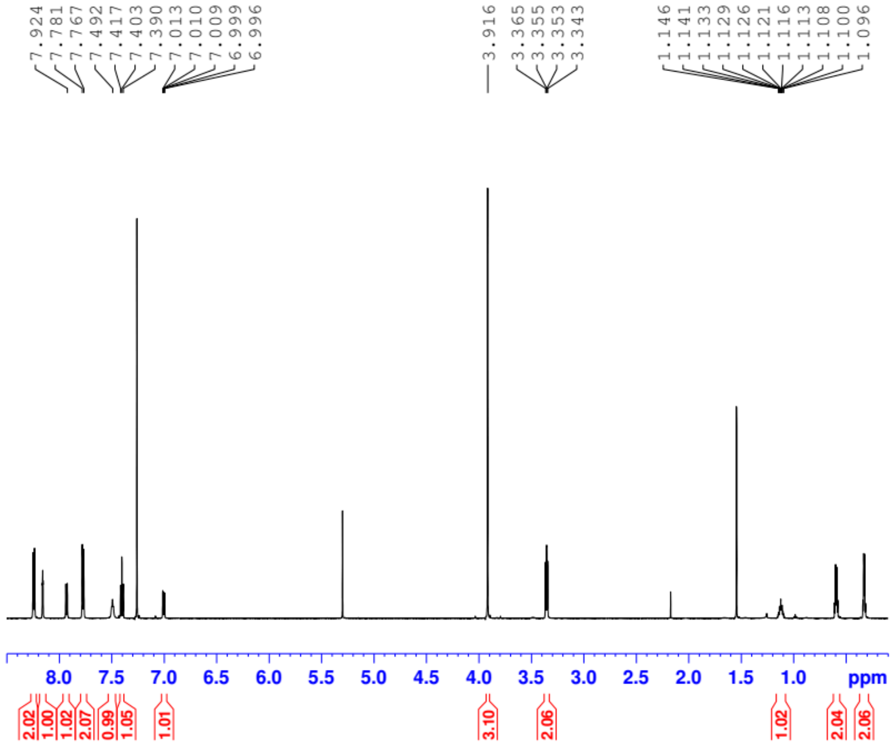

Figure S11. ^{1}H NMR spectra of compound **5k** (KB-2777) measured in $CDCl_3$ at 600 MHz

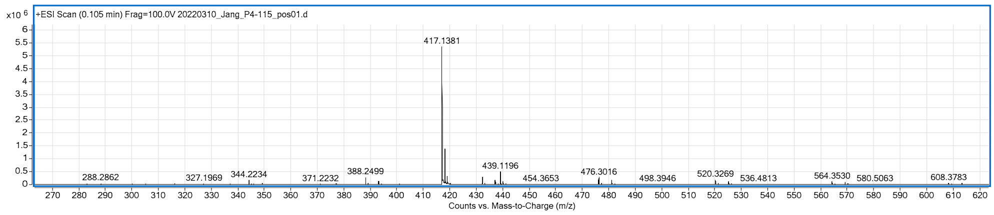

Figure S12. HRMS spectrum of compound **5k** (KB-2777)

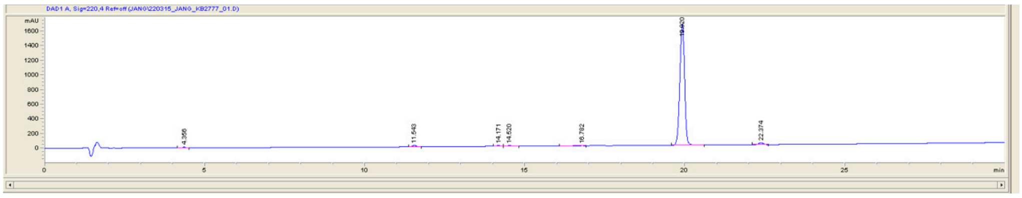

Figure S13. HPLC chromatogram of compound **5k** (KB-2777)

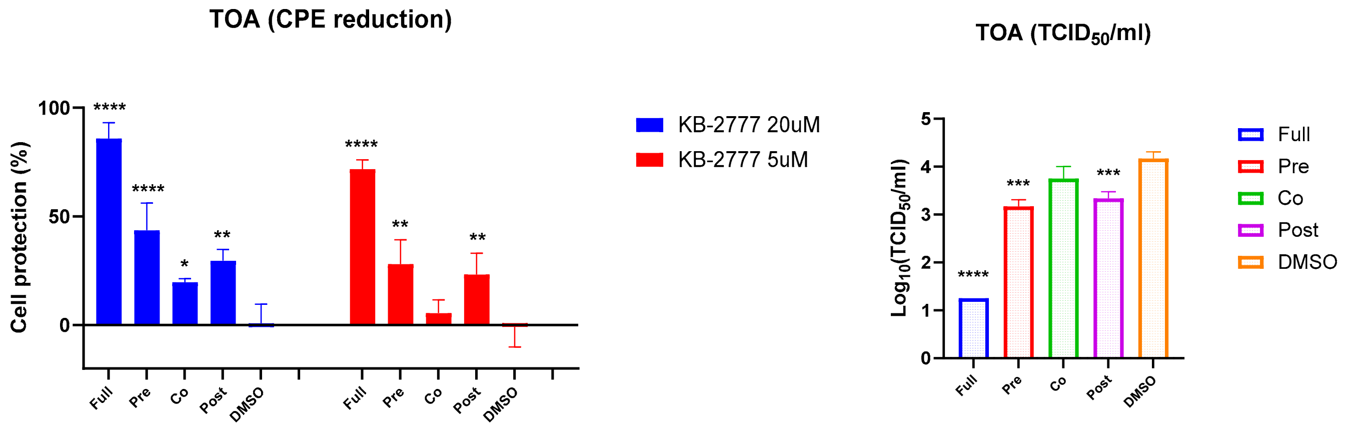

(a) (b)

Figure S14. Time-of-addition (TOA) analysis of KB-2777 against HCoV-NL63 (48 h). (a) CPE-reduction assay. KB-2777 was applied at 20 μM (blue) or 5 μM (red) under the indicated TOA conditions (Full, Pre, Co, Post, DMSO). Cell protection (%) was calculated relative to the DMSO control. Bars show mean ± SD (n = 3). Statistics: one-way ANOVA with Dunnett's multiple comparisons vs DMSO; *P<0.05, **P<0.01, ***P<0.001, ****P<0.0001. (b) Infectious progeny released under the same TOA conditions, quantified as log10($TCID_{50}$/mL) from culture supernatants (see Methods). Colors denote TOA conditions

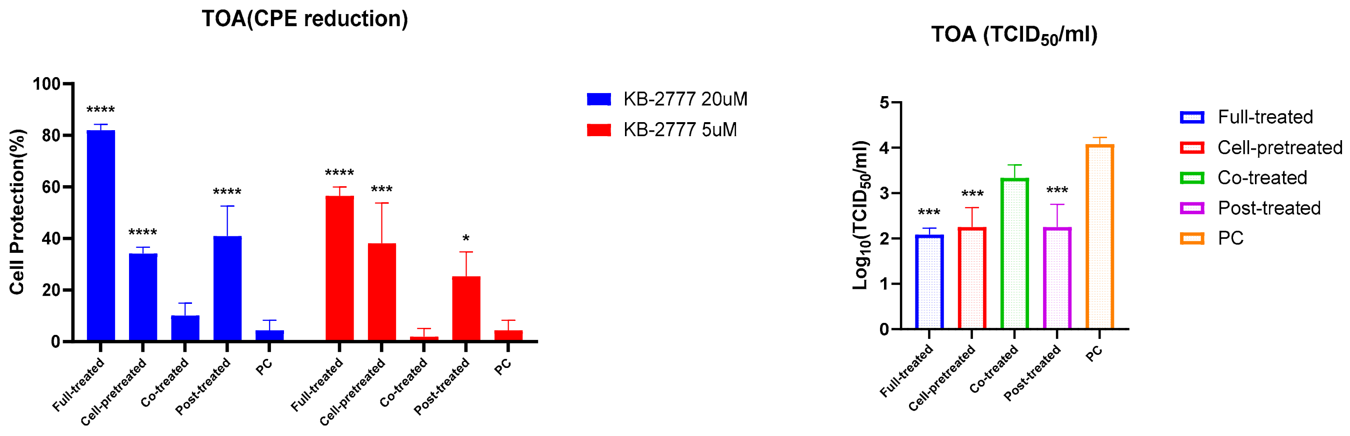

(a) (b)

Figure S15. Time-of-addition (TOA) analysis of KB-2777 against HCoV-OC43 (48 h). (a) CPE-reduction assay in MRC-5 cells. KB-2777 was applied at 20 μM (blue) or 5 μM (red) under the indicated TOA conditions (Full, Pre, Co, Post, DMSO). Cell protection (%) was calculated relative to the DMSO control. Bars show mean ± SD (n = 3). Statistics: one-way ANOVA with Dunnett's multiple comparisons vs DMSO; *P<0.05, **P<0.01, ***P<0.001, ****P<0.0001. (b) Infectious progeny released under the same TOA conditions, quantified as log_{10}($TCID_{50}$/mL) from culture supernatants by endpoint dilution on Vero E6 indicator cells (Spearman–Kärber method; see Methods). Points/bars show mean ± SD of n = 3 independent experiments; colors denote TOA conditions.

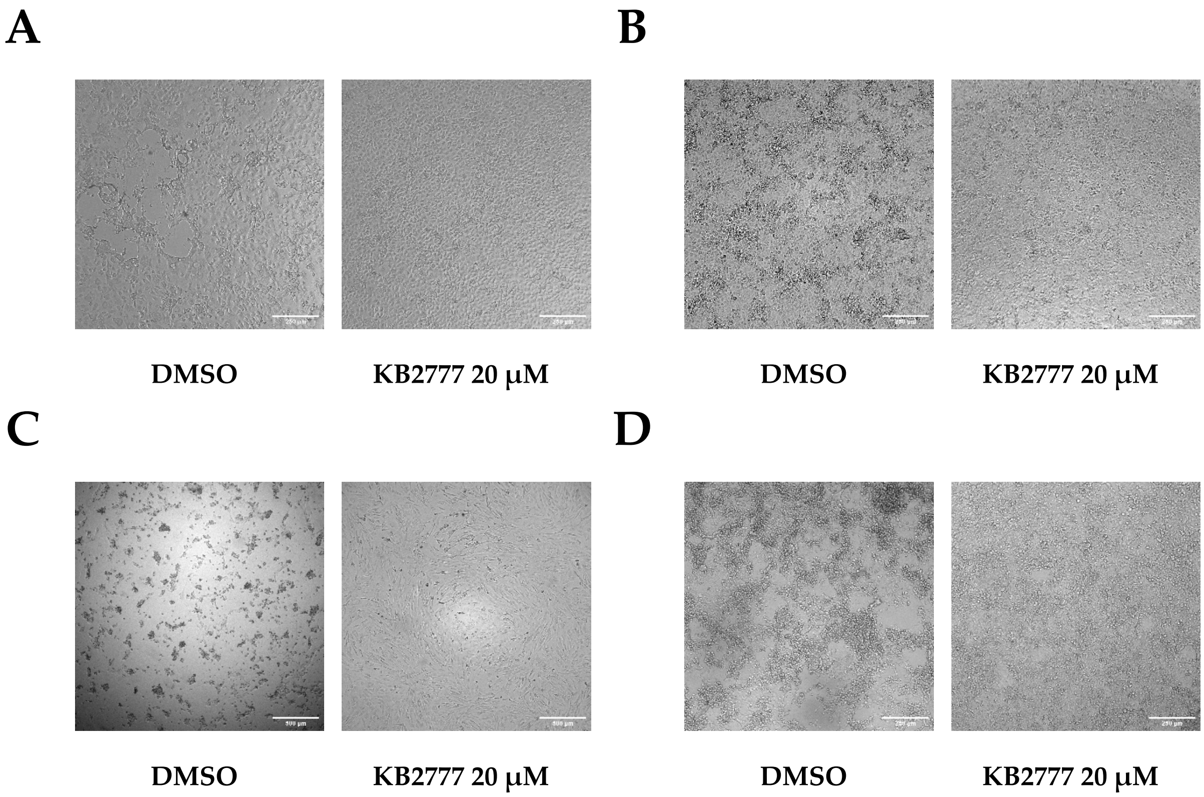

Figure S16. Cytopathic effect (CPE) protection by KB-2777 (20 μM) in coronavirus-infected cells. Cells were seeded 24 h before infection. Compound pretreatment was applied for 24 h, followed by 1 h virus adsorption, washing, and post-treatment with the same compound concentration. CPE was assessed at 72 h post-infection. (A) HCoV-NL63 in LLC-MK2 cells, (B) HCoV-OC43 in Vero E6 cells, (C) HCoV-OC43 in MRC-5 cells, and (D) PEDV in Vero E6 cells.

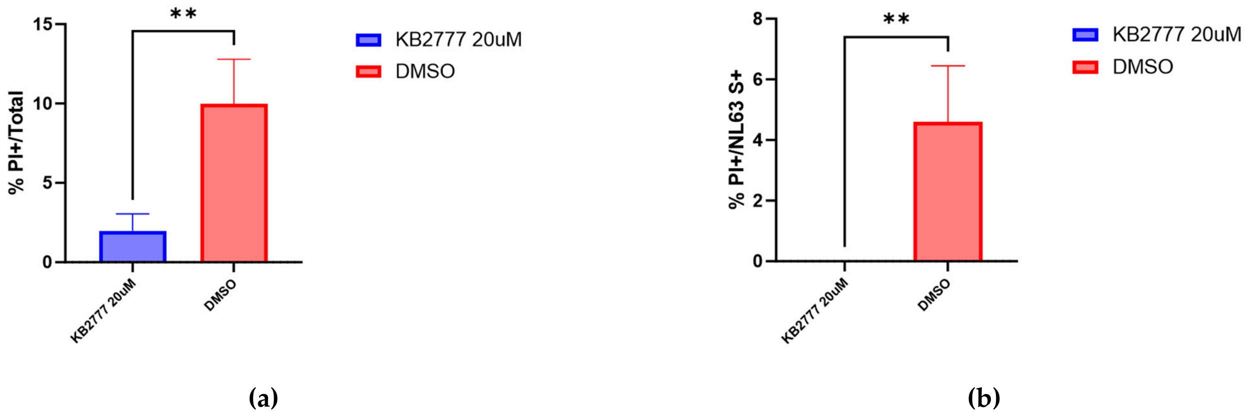

Figure S17. KB-2777 reduces PI-positive fractions in NL63-infected cultures (48 h post-infection) (a) % PI^+/Total cells. (b) % PI^+ among infected cells (PI^+/NL63 S^+). Each dot represents one field (n = 8 per group); bars indicate mean ± SEM. PC, virus + DMSO; KB-2777 20 μM, virus + KB-2777 (20 μM). Statistics used the Mann–Whitney U test (two-tailed); $p < 0.01$ for both comparisons.

Table S1. Antibodies for immunofluorescence (IF)

Antibodies	Source	Identifier
HCoV-NL63 Spike Antibody	R&D Systems	MAB11023-100
Goat Anti-Rabbit IgG H&L (FITC)	Abcam	ab6717

Table S2. Viral RT–qPCR primers/probes.

Target	Oligo	Sequence (5′→3′)
HCoV-OC43 M	**Forward**	ATG TTA GGC CGA TAA TTG AGG ACT AT
	**Reverse**	AAT GTA AAG ATG GCC GCG TAT T
	**Probe**	FAM- CAT ACT CTG ACG GTC ACA AT -BHQ1
HCoV-NL63 ORF1a	**Forward**	ACG TAC TTC TAT TAT GAA GCA TGA TAT TAA
	**Reverse**	AGC AGA TCT AAT GTT ATA CTT AAA ACT ACG
	**Probe**	FAM- ATT GCC AAG GCT CCT AAA CGT ACA GGT GTT -BHQ1
PEDV N	**Forward**	CGCAAAGACTGAACCCACTAACCT
	**Reverse**	TTGCCTCTGTTGTTACTTGGAGAT
	**Probe**	FAM- TGTTGCCATTACCACGACTCCTGC -BHQ1

Table S3. Human host-gene primers.

Gene name	Forward (5′→3′)	Reverse (5′→3′)	GenBank accession number
IL-6	GCA TGG GCA CCT CAG ATT GT	TGC CCA GTG GAC AGG TTT CT	NM_000600.5
ISG15	CTC TGA GCA TCC TGG TGA GGA A	AAG GTC AGC CAG AAC AGG TCG T	NM_005101.4

NQO1	CCT GCC ATT CTG AAA GGC TGG T	GTG GTG ATG GAA AGC ACT GCC T	NM_000903.3
HO-1	AAG ACT GCG TTC CTG CTC AAC	AAA GCC CTA CAG CAA CTG TCG	NM_002133.3
IFNB1	CTT GGA TTC CTA CAA AGA AGC AGC	TCC TCC TTC TGG AAC TGC TGC A	NM_002176.4
DDIT3(CHOP)	GGT ATG AGG ACC TGC AAG AGG T	CTT GTG ACC TCT GCT GGT TCT G	NM_004083.6
GCLM	TCT TGC CTC CTG CTG TGT GAT G	TTG GAA ACT TGC TTC AGA AAG CAG	NM_002061.4
XBP1	CCT TGT AGT TGA GAA CCA GG	GGG GCT TGG TAT ATA TGT GG	NM_005080.4
ATF4	TTC TCC AGC GAC AAG GCT AAG G	CTC CAA CAT CCA ATC TGT CCC G	NM_001675.4
ACTB	TGG ATC AGC AAG CAG GAG TAT G	GCA TTT GCG GTG GAC GAT	NM_001101.5
